# OH cleavage from tyrosine: debunking a myth

**DOI:** 10.1107/S1600577516016775

**Published:** 2017-01-01

**Authors:** Charles S. Bury, Ian Carmichael, Elspeth F Garman

**Affiliations:** a Laboratory of Molecular Biophysics, Department of Biochemistry, University of Oxford, South Parks Road, Oxford OX1 3QU, UK; b Notre Dame Radiation Laboratory, University of Notre Dame, Notre Dame, IN 46556, USA

**Keywords:** tyrosine, specific damage, radiation chemistry, electron density loss, Fourier difference maps

## Abstract

During macromolecular X-ray crystallography experiments, protein crystals held at 100 K have been widely reported to exhibit reproducible bond scission events at doses on the order of several MGy. With the objective to mitigate the impact of radiation damage events on valid structure determination, it is essential to correctly understand the radiation chemistry mechanisms at play. OH-cleavage from tyrosine residues is regularly cited as amongst the most available damage pathways in protein crystals at 100 K, despite a lack of widespread reports of this phenomenon in protein crystal radiation damage studies. Furthermore, no clear mechanism for phenolic C—O bond cleavage in tyrosine has been reported, with the tyrosyl radical known to be relatively robust and long-lived in both aqueous solutions and the solid state. Here, the initial findings of Tyr –OH group damage in a myrosinase protein crystal have been reviewed. Consistent with that study, at increasing doses, clear electron density loss was detectable local to Tyr –OH groups. A systematic investigation performed on a range of protein crystal damage series deposited in the Protein Data Bank has established that Tyr –OH electron density loss is not generally a dominant damage pathway in protein crystals at 100 K. Full Tyr aromatic ring displacement is here proposed to account for instances of observable Tyr –OH electron density loss, with the original myrosinase data shown to be consistent with such a damage model. Systematic analysis of the effects of other environmental factors, including solvent accessibility and proximity to di­sulfide bonds or hydrogen bond interactions, is also presented. Residues in known active sites showed enhanced sensitivity to radiation-induced disordering, as has previously been reported.

## Abstract 2

A systematic macromolecular crystallography investigation into the observed electron density loss around the –OH group of tyrosines, as a function of dose at 100 K, is reported. It is concluded that a probable explanation is aromatic ring disordering as opposed to –OH cleavage; occurrence of the latter mechanism is a misconception perpetuated in radiation damage literature, and is unsupported by any observations in radiation chemistry.

## 1. Introduction   

The effects of radiation damage incurred during data collection continue to impede successful structure determination of proteins by macromolecular X-ray crystallography (MX), even at cryo temperatures (Garman & Weik, 2015 ▸). Global damage is manifest in the fading of the diffraction pattern with those reflections pertinent to high-resolution diminishing first, principally as a result of increasing crystal non-isomorphism (Murray & Garman, 2002 ▸). Multiple global damage *dose limits* have been proposed as upper bounds for the accumulated dose required to reduce the diffractive power of any macromolecular crystal below a specified fraction (typically 50%). These MX dose limits appear consistent across studies; indeed the originally reported half-dose (*D*_0.5_ = 20 MGy), theoretically derived by comparison with two-dimensional electron diffraction experiments at 77 K (Henderson, 1990 ▸), is on the same order as subsequent, experimentally derived values: *D*_0.5_ = 43 MGy (Owen *et al.*, 2006 ▸) and, for a resolution of *s* Å, a resolution-dependent dose limit of *D*_0.5_ = 10 × *s* MGy (Howells *et al.*, 2009 ▸). An experimental dose limit has been determined as 30 MGy at cryo-temperatures (typically ∼100 K), corresponding to the total recorded intensity dropping to 0.7 of its original value, beyond which biological information may well be compromised (Owen *et al.*, 2006 ▸). These upper limits appear to be constrained by the associated physics, although chemical effects, so-called specific damage, have been observed at much lower doses, with damage reportedly occurring in a well defined order across a broad range of proteins. Specific damage is typically observed by reconstructing electron density maps, more precisely *F*_obs_(*n*) − *F*_obs_(1) difference maps, to monitor changes to electron density as dose increases. Already, at doses of the order of 0.35 MGy, redox-active metal centres are reduced (Corbett *et al.*, 2007 ▸) and, as the dose increases, apparent damage, *i.e.* areas of negative difference electron density, have been reported around di­sulfide bonds, side-chain carb­oxy­lic groups on aspartates and glutamates, the OH group on tyrosines and around the C—S bonds of me­thio­nines (Burmeister, 2000 ▸; Weik *et al.*, 2000 ▸; Ravelli & McSweeney, 2000 ▸).

Extensive radiation chemical studies in aqueous solution have shown that di­sulfide bonds are susceptible to both oxidation, for example by hydroxyl radicals, and reduction by the associated solvated electrons. A convincing demonstration that the latter mechanism prevails in lysozyme crystals X-irradiated at cryo-temperatures has been published (De La Mora *et al.*, 2011 ▸). In that work, the complementary technique of microspectrophotometry revealed changes in optical absorption as a function of dose, with a peak around 500 nm, associated with radiation-induced trapped electrons, diminishing in concert with the growth of a peak around 400 nm. The latter band could be reliably attributed to the formation of a di­sulfide radical anion, in which the S—S internuclear distance is substantially increased from that in the neutral di­sulfide residue, a feature visible in reconstructed electron densities as a function of increasing dose (Weik *et al.*, 2002 ▸).

Oxidative de­carboxyl­ation of amino acids is also well known from aqueous radiation chemistry, where electron loss in the zwitterionic residue leads to C_α_—CO_2_ bond lengthening at the C-terminus and eventual loss of CO_2_ (Wisniowski *et al.*, 2002 ▸). In MX, side-chain de­carboxyl­ation is also seen in aspartates and glutamates, with quantum chemical calculations indicating that the process is more thermodynamically favourable in the latter (Bury *et al.*, 2016 ▸).

Additionally, me­thio­nine residues are also readily oxidized under radiation chemical conditions (Kopoldova *et al.*, 1967 ▸). The me­thio­nine sulfur-centred radical cations which are formed predominantly enter irreversible reaction channels. These ultimately yield carbon-centred and/or peroxyl radicals, which are the starting points for chain reactions of protein oxidation (Schöneich, 2005 ▸). Cleavage of the C_β_—S bond is thus well documented.

Conversely, though the tyrosine residue is well known to be redox active, cleavage of the phenolic C—O bond has not been reported in the proposed mechanisms of tyrosine radiation chemistry. It was thus surprising that the radiation damage study of Burmeister (2000 ▸) provided such a clear display of electron density loss, and thus specific radiation damage, around the phenolic oxygen on a tyrosine residue during diffraction data collection from X-irradiated myrosinase crystals (Burmeister, 2000 ▸). Indeed the redox couple involving tyrosine and the tyrosyl radical plays a key role in high potential redox catalysis for a wide range of natural enzymes including, for example, photosystem II (Rappaport & Diner, 2008 ▸), ribonucleotide reductase (Stubbe *et al.*, 2003 ▸), cyctochrome *c* oxidase (Kaila *et al.*, 2010 ▸), galactose oxidase (Whittaker, 2003 ▸) and prostaglandin synthase (Tsai & Kulmacz, 2010 ▸), suggesting a robustness of the phenolic linkage.

Extensive experience from the aqueous radiation chemistry of tyrosine-containing solutions at room temperature also suggests the relative persistence of the tyrosyl radical. For example, from pulse radiolysis studies it is clear that attack by the strongly oxidizing hydroxyl radical, which is formed by the radiolysis of solvent water, leads predominantly to substitution on the aromatic ring. In the presence of oxygen, the eventual products are 2,4- and 3,4-di­hydroxy­phenyl­alanines (Chrysochoos, 1968 ▸; Solar *et al.*, 1984 ▸). Minor pathways involve ring–ring dimerization and H-abstraction from the β-carbon (Solar *et al.*, 1984 ▸). Other, less potent, one-electron oxidants initially lead to the formation of the tyrosyl radical, opening pathways leading to products which have recently been thoroughly reviewed (Houée-Levin *et al.*, 2015 ▸). In the solid state, soft X-ray irradiation of the respective powders showed phenyl­alanine degrading more rapidly than tyrosine (Zubavichus *et al.*, 2004 ▸). Also, γ-radiolysis at low temperature cleaves the former to give a benzyl radical, but again produces the tyrosyl radical in the latter (Fasanella & Gordy, 1969 ▸).

The original Burmeister (2000 ▸) study reported progressive accumulation of negative *F*_obs_(*n*) − *F*_obs_(1) difference density centred on the –OH group at active site residue Tyr-330 in myrosinase, coupled with observable positive difference density in the adjacent solvent region. In addition, a decaying –OH group occupancy with increasing dose was reported. Myrosinase functions to hydrolyze glucosinolates predominantly in the *Brassicaceae* family, with Tyr-330 situated in a glucose binding site and exhibiting a hydrogen bond to an acidic residue (Glu-409) in the absence of bound glucose (Burmeister *et al.*, 1997 ▸). Tyr-330 is predicted to reorientate to accommodate glucose insertion, with the Tyr hydroxyl group then positioned adjacent to the sugar ring oxygen (with no possibility to form a hydrogen bond to Glu-409). In the Burmeister (2000 ▸) study, an ordered glycerol molecule instead occupies the glucose-binding site, and Tyr-330 initially exhibits the hydrogen bond to Glu-409. Glu-409 was observed to undergo radiation-induced de­carboxyl­ation, leading Bur­meister to postulate the existence of a correlation between Tyr –OH damage and such hydrogen bonding interactions. The loss of the Tyr-330-stabilizing hydrogen bond would permit increased lability of the Tyr-330 aromatic ring, and would manifest itself in *F*_obs_(*n*) − *F*_obs_(1) maps mostly clearly at positions furthest from the Tyr C_β_ hinge-point (*i.e.* at the Tyr –OH group). Burmeister also suggested the possibility that an –OH cleavage event had occurred [legend Fig. 3(*c*), Bur­meister (2000 ▸)].

However, despite the ambiguity in the reason behind Burmeister’s finding of density loss around the tyrosyl oxygen, and the weight of evidence from radiation chemistry studies for tyrosyl persistence, such Tyr –OH electron density loss has subsequently been interpreted as OH loss for protein crystals at 100 K. Tyr –OH cleavage is now regularly cited [including by some of the current authors, see for example Garman (2010 ▸); Dubnovitsky *et al.* (2005 ▸); Owen & Sherrell (2016 ▸)] as one of the four dominant non-metalloprotein specific damage events present in MX experiments conducted at 100 K, despite there being no reported mechanism for phenolic C—O bond cleavage. Conversely, other than for myrosinase, to our knowledge there have been no other reports of clear Tyr –OH damage for protein crystal systems irradiated at 100 K.

To better characterize the reproducibility of Tyr –OH group electron density loss in irradiated protein crystals, we have performed a systematic investigation on a wide range of structures at 100 K. A subset of previously conducted MX radiation damage investigations have included deposition of readily accessible coordinate and structure factor information in the Protein Data Bank (PDB), providing an ideal database of protein damage series. Each damage series has been refined independently by different research groups, mitigating the risk of systematic and subjective errors in the refinements between different structures. Moreover, the deposited damage series have all *a priori* satisfied the PDB validation criteria, and can thus be deemed of suitable quality for the current study.

For the analysis of multiple MX damage series for distinct protein structures, a systematic visual interpretation of *F*_obs_(*n*) − *F*_obs_(1) maps would be impractical, with a high risk of subjective bias by the investigators. The *D*_loss_(atom) metric provides an objective dose-dependent atom-by-atom quantification of electron density loss. *D*_loss_ is derived from *F*_obs_(*n*) − *F*_obs_(1) difference maps at each subsequent accumulated dose (*n* = 2,…) as the maximum density loss value in the local vicinity of each atom [for full construction see Bury *et al.* (2016 ▸)]. Here, *F*_obs_(*n*) − *F*_obs_(1) difference maps are calculated from the differences of reflection amplitudes between the first and each successive data set and with a fixed phase set, *φ*_calc_(1), derived from model refinement against the first data set. Our *D*_loss_ metric was originally introduced to enable analysis of the distribution of specific damage sites within a large (∼204 kDa) bacterial protein-RNA complex (Bury *et al.*, 2016 ▸).

This paper reports the results of an investigation into tyrosine electron density loss in the available damage series using this *D*_loss_ metric.

## 2. Materials and methods   

### 2.1. Reanalysis of the myrosinase damage series (Burmeister, 2000 ▸)   

To assess the quality of the originally deposited myrosinase damage series (Burmeister, 2000 ▸), the coordinate and 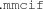 structure factor files were retrieved from the PDB (accession codes: 1dwa, 1dwf, 1dwg, 1dwh, 1dwi, 1dwj). For the original publication, two coordinate models (PDB: 1dwi and 1dwj) had been deposited for the highest dose state, corresponding to occupancy and positional refinement, respectively. The 1dwi coordinate model was used for the current analysis. For comparison, coordinate and structure factor files were extracted from *PDB_REDO* (Joosten *et al.*, 2014 ▸), both after initial file curation [ten cycles of rigid body refinement in *REFMAC* (Murshudov *et al.*, 2011 ▸)] and after full model re-building. Overall atomic fits to electron density [real-space *Z*_obs_ scores and real-space correlation coefficient (RSCC)] were computed using *EDSTATS* (Tickle, 2012 ▸).

For the initial coordinate model (PDB: 1dwa) of the Burmeister (2000 ▸) damage series retrieved from *PDB_REDO* following full model re-building, a secondary Tyr-330 side-chain conformation was built manually in *COOT* (Emsley *et al.*, 2010 ▸). Positive difference density in the *F*_obs_(4) − *F*_obs_(1) map nearby the Tyr-330 aromatic ring (contoured at ±5σ) was used as a guide for suitable placement. To assess the conformational variability of Tyr-330 with dose, for each dataset within this damage series, occupancy refinement was performed in *phenix.refine* (Adams *et al.*, 2010 ▸) with atomic *B*-factors held constant, using this modified coordinate model coupled with the structure factor amplitudes for that dataset. The presence of unmodelled alternate Tyr side-chain conformations at each dose was detected using *RINGER* (Lang *et al.*, 2010 ▸). *Bdamage* (Gerstel *et al.*, 2015 ▸) was calculated for myrosinase as a quantifier for radiation-induced flexibility of Tyr aromatic side groups. Prior to this calculation, to account for the dependence of *Bdamage* on non-unity atomic occupancies and reported atomic *B*-factor values, all occupancies in each myrosinase coordinate model were set to 1 and a further round of isotropic *B*-factor refinement was performed in *phenix.refine*.

### 2.2. *PDB_REDO* damage series analysis   

A complete (to our knowledge) list of MX damage series with both coordinate models and structure factor files deposited in the PDB was collated. Of these, a selection was made using criteria designed to ensure a suitable sample set for the current analysis: (*a*) only MX experiments explicitly conducted at 100 K were included, (*b*) studies with reported accumulated doses <1 MGy (at highest dose dataset) were omitted altogether, and (*c*) intermediate datasets with reported total doses <1 MGy within the remaining series were excluded. A 1 MGy lower limit was imposed since the first signs of specific damage to metal-free protein crystals at 100 K (di­sulfide radicalization and cleavage) have been detected in *F*_obs_(*n*) − *F*_obs_(1) maps below 1 MGy, whereas Glu/Asp de­carboxyl­ation has not (Sutton *et al.*, 2013 ▸). Datasets (apart from the first one) with doses below 1 MGy were thus deemed unsuitable for the purpose of investigating possible Tyr –OH damage events. The complete Sutton *et al.* (2013 ▸) lysozyme damage series (reported dose range 0.07–1.05 MGy) was additionally included as a control in order to justify the chosen 1 MGy lower bound. Table 1 ▸ provides a full list of the 16 damage series located in the PDB that were used in the current study.

For each MX damage dataset series, PDB-format coordinate model and MTZ-format structure factor files were retrieved from the *PDB_REDO* online server. For each damage series, files were retrieved after initial *PDB_REDO* file curation, but prior to any further model rebuilding. At this stage, coordinate models had been subjected to either 0 or 10 cycles of rigid body refinement with *REFMAC*, dependent on whether *PDB_REDO* flagged possible *R*_free_ bias within the PDB-deposited data. This procedure was chosen to extract data as close to that used by the authors of the associated radiation damage studies as possible, whilst ensuring that all data were in a consistent format. Accordingly, for the initial structure in each damage series, with the exception of myrosinase, the recalculated *R*_work_ statistics were all reported by *PDB_REDO* to be within 2% of those reported in the originally deposited coordinate file header (and for structures where there was no *PDB_REDO* flagged potential *R*_free_ bias, *R*_free_ was also within 1% of the original reported values), see Table S1.1 of the supporting information for details. For the myrosinase damage series (Burmeister, 2000 ▸), the coordinate model following full model rebuilding with *PDB_REDO* was used in the current analysis, since the *2mF*_obs_ − *DF*_calc_ map-model fit was poor for the original PDB-deposited 1dwa model (see §3.1 for details).

Further rigid body refinement was performed in *REFMAC* for each higher dose dataset, now using the coordinate model from the initial dataset coupled with the structure factor amplitudes corresponding to the higher dose dataset. This ensured that an identical set of atoms was modelled for each dataset within a damage series, whilst accounting for whole-molecule translation and rotation within the unit cell, as a result of any changes in unit-cell dimensions with increasing dose. The resulting coordinate models were used throughout the analysis presented here.

It must be emphasized that the reported maximum attained resolution differs between each protein system (from 1.2 to 2.8 Å). The optimum model refinement strategy (*e.g.* applicability of NCS, *B*-factor restraints) is very much dependent on resolution, and it is recognized that the selected proteins have undergone non-uniform refinement protocols prior to retrieval. Consequently, a *B*-factor-derived metric [*e.g.* relative atomic *B*-factor increase (Weik *et al.*, 2000 ▸) or *Bdamage* (Gerstel *et al.*, 2015 ▸)] was deemed unsuitable for comparison between damage series. For each MX damage series, a per-atom quantification, *D*_loss_(atom), of the radiation-induced electron density loss derived from *F*_obs_(*n*) − *F*_obs_(1) Fourier difference maps (*n* = 2,3,…), was computed at each dose using the program *RIDL* (available upon request from the authors). A metric derived from *F*_obs_(*n*) − *F*_obs_(1) maps depends directly on experimentally determined diffraction intensities, thus minimizing dependence on refined models. *F*_obs_(*n*) − *F*_obs_(1) maps were generated using *FFT* (Ten Eyck, 1973 ▸) as part of the *RIDL* pipeline. Initial manual inspection of maps was performed in *COOT* to ensure that key specific damage manifestations (*e.g.* clear negative difference density surrounding di­sulfide bonds) were consistent with reports in the original publications pertaining to the particular damage series. Per-atom solvent accessibilities were calculated with the CCP4 program *AREAIMOL*, accounting for crystal contacts between adjacent asymmetric units, and hydrogen bonding interactions within structures were determined using *NCONT* (Winn *et al.*, 2011 ▸).

## 3. Results   

### 3.1. Reanalysis of the myrosinase damage series (Burmeister, 2000 ▸)   

For the first dataset in the myrosinase series (PDB accession code: 1dwa), *PDB_REDO* reported poor agreement between *R*_work_/*R*_free_ given by the 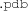 header originally deposited in the PDB (0.165/0.179) and those recalculated directly from the deposited data post rigid body refinement but prior to model rebuilding (0.191/0.201). Visual inspection of the 2*mF*_obs_ − *DF*_calc_ map retrieved from the Electron Density Server (EDS) revealed regions of poor fits of this model for ordered solvent and side-chain conformations to density (overall map RSCC: 0.67), including several Tyr aromatic groups (notably Tyr-215). To recalculate *R*_work_/*R*_free_ from the original 1dwa data, *PDB_REDO* carried out a default 0-cycle *REFMAC* refinement, followed by 10-cycles of rigid body refinement (standard procedure in *PDB_REDO* when the *R*_work_/*R*_free_ reported in the original 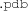 header are not reproducible to within 5% after the single 0-cycle *REFMAC* run). The 10-cycle rigid body refinement of 1dwa was observed to improve the fit of Tyr side-chains to density (with overall map RSCC: 0.920). Corresponding real-space EDSTATS *Z*_obs_ scores both pre- and post- further rigid body refinement are provided in Table S1.2 of the supporting information. Further full model rebuilding in *PDB_REDO* again improved the refinement statistics (*R*_work_/*R*_free_: 0.13/0.16 with RSCC: 0.97). The improvement in the Tyr-215 electron density fit is shown in Fig. S1.1 of the supporting information.

**Figure 1:**
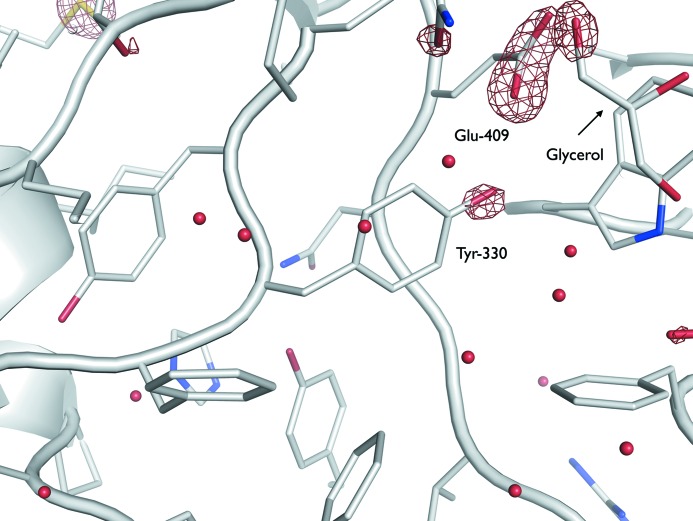
Specific radiation damage to Tyr-330 and Glu-409 in the myrosinase damage series. *F*_obs_(5) − *F*_obs_(1) difference map, contoured at ±5σ in green/red, overlaid on the initial coordinate model in white (PDB accession code: 1dwa). Coordinate model and structure factor amplitudes were retrieved from *PDB_REDO*, and *F*_obs_(5) − *F*_obs_(1) maps were generated by *FFT* within the *RIDL* pipeline. Electron density loss is observable around a glycerol oxygen, presumably also due to the dislocation of the glycerol upon de­carboxyl­ation of Glu-409.

**Figure 2:**
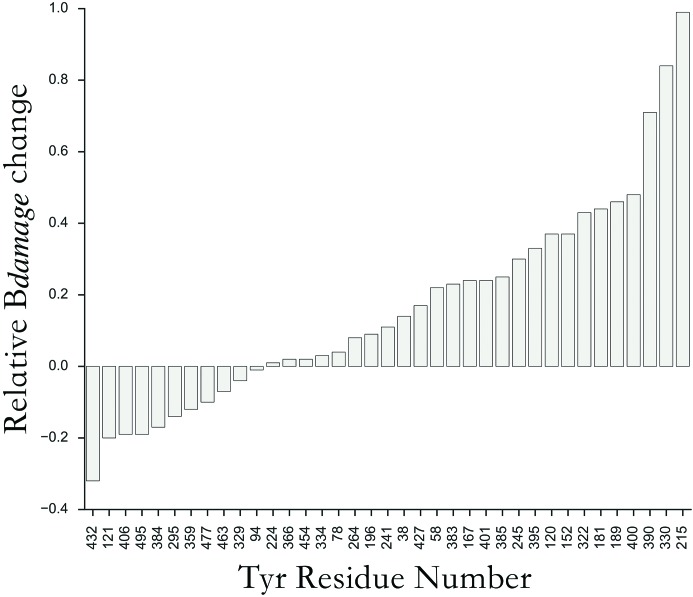
*Bdamage* change for each Tyr –O atom in the highest dose myrosinase structure (PDB accession code: 1dwi) relative to the 1dwa structure. For each atom *a*, the relative change is calculated as [*Bdamage*(*a* ^1dwi^) − *Bdamage*(*a* ^1dwa^)]/*Bdamage*(*a* ^1dwa^). To account for non-unity occupancies in the originally deposited data, all atomic occupancies in the 1dwa and 1dwi coordinate models were set to 1 and a further round of isotropic *B*-factor refinement was performed in *phenix.refine* prior to calculating *Bdamage*.

Consistent with Burmeister’s original findings, interpretable negative difference density in *F*_obs_(*n*) − *F*_obs_(1) maps located at the Tyr-330 –OH group was observed above (*i.e.* more negative) a −5σ map threshold for the highest dose datasets (*n* = 4, 5) (Fig. 1 ▸). An additional negative difference peak was coincident with the Tyr-215 –OH group (also clear above −5σ for *n* = 4, 5), a finding not reported in the original study (Fig. S1.2). We suggest that in that work the poor density fit for Tyr-215 resulted in no difference peak being attributed to Tyr-215. Indeed, upon our re-inspection of the *F*_obs_(5) − *F*_obs_(1) map from the original data extracted from the PDB, a nearby difference peak was revealed; however, it was not directly aligned with the Tyr-215 –OH group (Fig. S1.2a). Significant density loss peaks (apparent at > −4σ) were also identifiable in the *F*_obs_(5) − *F*_obs_(1) map centred around several other Tyr –OH groups (Tyr-390 above −5σ, and Tyr-400, Tyr-181 and Tyr-152 above −4.5σ). Both the relative atomic *B*-factor increase and *Bdamage* atomic displacement metrics exhibited strong agreement with these observations, with Tyr-330, Tyr-215 and Tyr-390 flagged as highly sensitive compared with other Tyr –OH groups (Fig. 2 ▸). Here the *Bdamage* metric provides a quantification of increased residue flexibility with dose, deconvoluted from atomic packing density.

Overall, although *PDB_REDO* provided a significant improvement in *R*_work_/*R*_free_ statistics for the original initial myrosinase structure 1dwa, the damage signatures reported by Burmeister (2000 ▸) could be replicated.

### 3.2. Investigation of radiation-induced trends between proteins   

In the publications relating to previous radiation damage investigations, dose values have been independently calculated using a variety of tools [*e.g. RADDOSE* v1 (Murray *et al.*, 2004 ▸), v2 (Paithankar *et al.*, 2009 ▸) and v3 (Paithankar & Garman, 2010 ▸), and *RADDOSE-3D* (Zeldin *et al.*, 2013*a* ▸)]. As such, direct comparison of raw *D*_loss_ values between protein crystals *reported* to be at identical doses is highly sensitive to miscalibration between dose scales. No attempt has been made here to recalculate doses for the damage series, and all doses are as reported within the original publications. A discrete ranking scheme has been implemented to mitigate uncertainties when comparing density map-derived metric values between independent protein damage series, since experimental procedures and refinement strategies are also not conserved amongst them.

For a damage series consisting of a single low- and high-dose dataset, *D*_loss_ values correspond to the single *F*_obs_(2) − *F*_obs_(1) map, and each atom type *A* (*e.g.* the set of Tyr –OH oxygen atoms) is ranked with respect to the mean *D*_loss_ values attained by that atom type (rank = *R*_A_ ≥ 0). For damage series containing multiple higher dose datasets, atom type ranks have been determined per-dataset (*R*_A,*i*_ for *i* = 2,…, *n*) and the products of these per-dataset ranks (*R*_A,2_ × *R*_A,3_ × *…* × *R*_A,*n*_) are then ranked to provide an across-dataset rank for each atom type.

**Figure 3:**
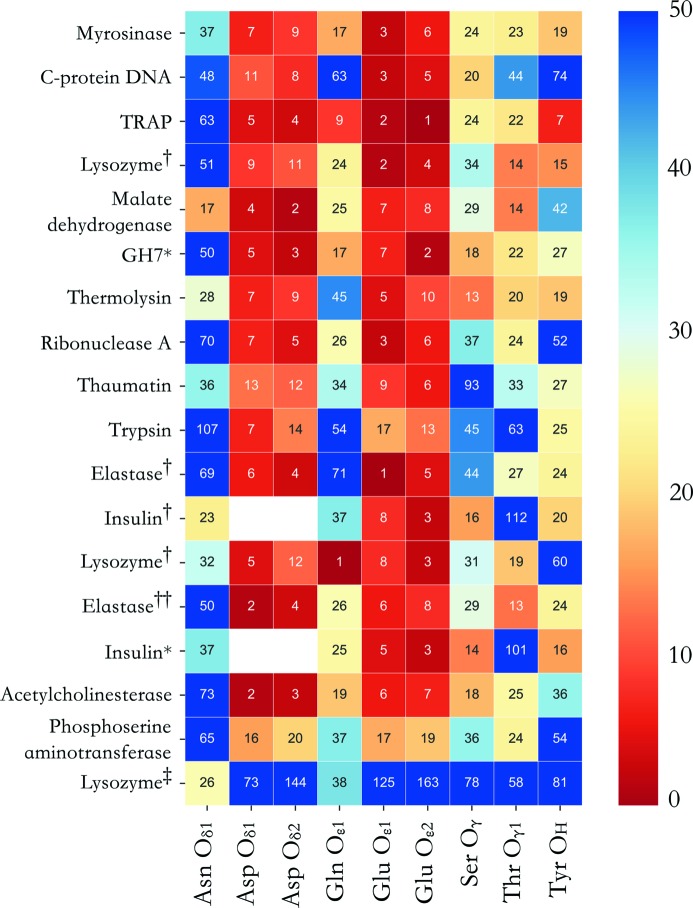
Density loss ranking for each damage series for distinct side-chain oxygen atom types occurring within each protein structure. Blank squares represent atom types not present within a given protein. The ranking scheme starts at 0 for the most damaged atom species within each protein, thus a large ranking number indicates low *D*_loss_. For clarity, each square is coloured by rank, with the corresponding colour key provided to the right of the grid. Superscripted proteins correspond to the following studies: †Nanao *et al.* (2005 ▸), ††Petrova *et al.* (2010 ▸), ‡Sutton *et al.* (2013 ▸). *Data collected from GH7 and insulin crystals (distinct from *PDB_REDO* collated series) are additionally included as part of the test set (see Table 1 ▸ for details).

Fig. 3 ▸ illustrates the resulting ranks of radiation-induced electron density loss for different side-chain oxygen moieties in the damage series investigated. Only the oxygen atoms in the structures have been compared with Tyr –OH oxygen, to maintain a constant total number of electrons per compared atom. In a protein at a specific dose, neglecting variations in thermal motions between different side-chains, two oxygen atoms O_A_ and O_B_ should exhibit identical *D*_loss_(atom) values if the yields of O_A_ and O_B_ cleavage events throughout the protein crystal are the same. Whereas clear Glu and Asp de­carboxyl­ations are indicated by uniformly high Glu-O_∊1_/O_∊2_ and Asp-O_δ1_/O_δ2_ ranks, Tyr –O is consistently ranked lower, and of a similar order as seen in the control group of Gln, Asn, Ser and Thr O atoms for each protein structure, which are not suspected to be cleaved. Indeed in 14/18 damage series, at least one out of the control group has a rank higher than that of Tyr –O. In particular, for the myrosinase series (Burmeister, 2000 ▸), Gln-O_∊1_ receives a higher ranking than Tyr –O.

In phospho­serine amino­transferase, Glu and Asp are ranked as less susceptible than typically observed, with the highest rankings instead attained by atoms within the lysine-pyridoxal-50-phosphate Schiff base present within the protein (data not shown). Indeed, the Schiff base active site was reported in the original publication (Dubnovitsky *et al.*, 2005 ▸) to undergo severe radiation-induced relaxation of chemical strain, with pronounced reorientation of the Schiff base cofactor. The poor rankings (>73, Fig. 3 ▸) for Glu and Asp in lysozyme (Sutton *et al.*, 2013 ▸) are consistent with the low maximum dose (1.05 MGy) reported for this damage series. Whereas di­sulfide bond breakage is reported in this study, the dose is below the onset of Glu/Asp de­carboxyl­ation for this protein.

Overall it is evident that despite unavoidable variability between the included protein systems (*e.g.* differing maximum resolution, crystallization buffers), the implemented *D*_loss_ ranking scheme is capable of detecting specific damage trends throughout the investigated proteins. Regardless of the feasibility of a radiation-induced Tyr –OH damage event within crystalline protein at 100 K, Tyr –OH density loss is in general significantly lower than for Glu and Asp carboxyl­ates for doses >1 MGy, at which de­carboxyl­ation events were reported.

### 3.3. A hypothesis for the observed Tyr electron density loss   

**Figure 4:**
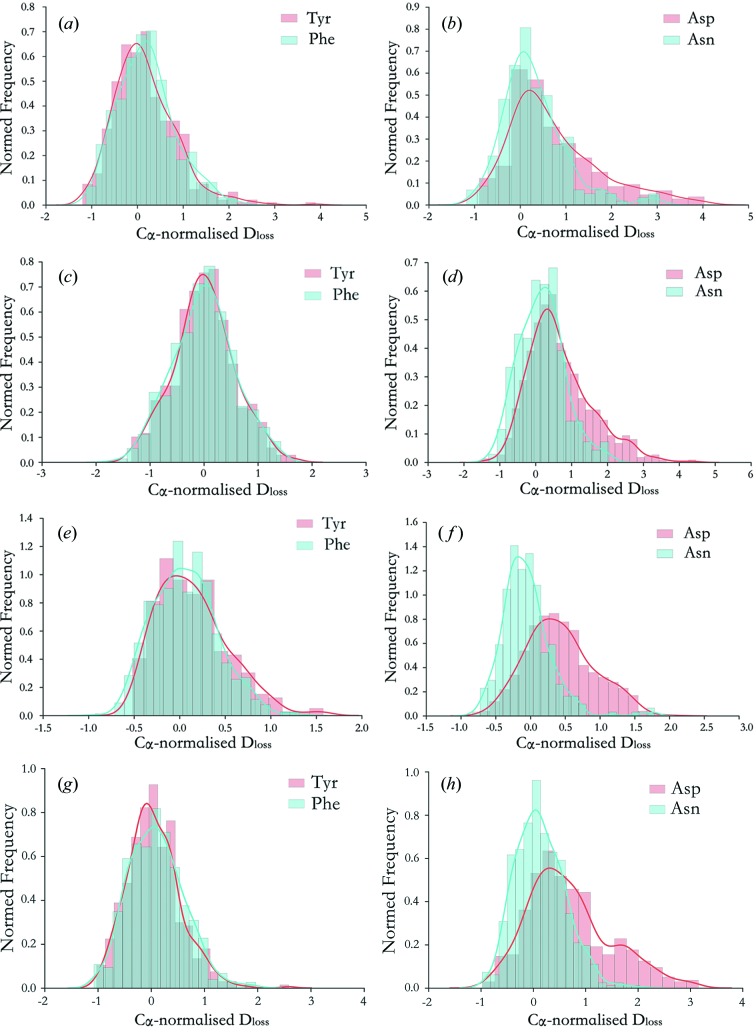
C_α_-normalized *D*_loss_ damage signature histogram plots over all atoms of specific residue types for (*a*, *b*) myrosinase, (*c*, *d*) malate de­hydrogenase, (*e*, *f*) TRAP and (*g*, *h*) GH7. The normalization scheme is: C_α_-normalized *D*_loss_(atom) = [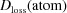 − 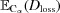]/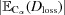, where 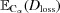 is the average 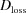 calculated over the set of C_α_ atoms. Two residue types are directly compared in each plot: (*a*, *c*, *e*, *g*) Tyr and Phe, and (*b*, *d*, *f*, *h*) Asp and Asn. Gaussian kernel density estimates, which are non-parametric approximations of the probability density function underlying each *D*_loss_ distribution, are overlaid on the histogram plots. Highest dose datasets for each series are displayed; other doses within each series exhibit qualitatively similar behaviour.

**Figure 5:**
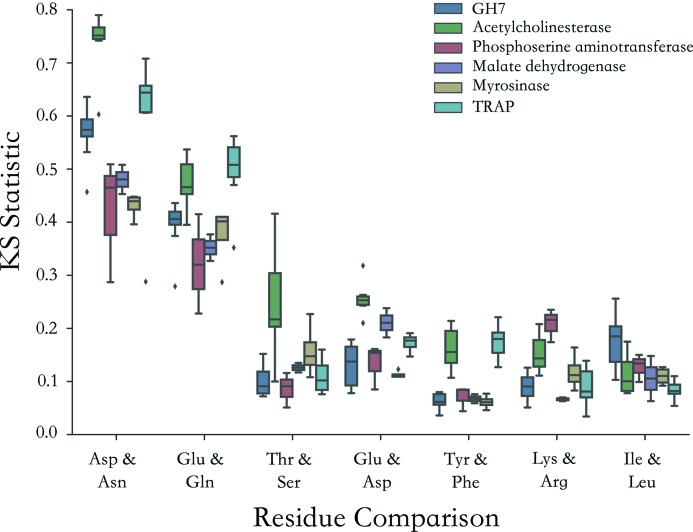
Kolmogorov–Smirnov (KS) test statistics comparing similarity of different residue types for large protein structures (>60 kDa). For each damage series, box plots illustrate the distribution in KS statistic values across all the datasets in that series. A higher KS statistic indicates less similarity between the distribution of *D*_loss_ values for a specified residue pairing.

Here the distribution of *D*_loss_ values (so-called *damage signatures*) for topologically similar residue types have been directly compared. These are shown in Fig. 4 ▸ for the highest dose datasets for myrosinase, Trp RNA-binding attenuation protein (TRAP), malate de­hydrogenase (Fioravanti *et al.*, 2007 ▸) and the GH7 protein, all of which contain a statistically sufficient sample size of atoms for histogram analysis (each >62 kDa). For each residue, the damage signature plot contains *D*_loss_ information for all non-hydrogen atoms of that residue at a specific dose. In Fig. 4 ▸, at each dose, *D*_loss_ values have been normalized to the average *D*_loss_ value attained at that particular dose by the set of C_α_ backbone atoms in the structure. This quantifies per-atom density loss relative to a known radiation-insensitive set of atoms. As a result, signatures are observed to be typically unimodal distributions, with undamaged atoms attaining near zero values and right-tails indicating the presence of radiation-sensitive atom types within a residue. For each residue pairing, the Kolmogorov–Smirnov (KS) test has been employed as a non-parametric statistic to test the null hypothesis that the *D*_loss_ values of both residue types have been drawn from the same distribution. The KS-test statistic thus provides a quantifier for the similarity between the *D*_loss_ signatures for two residue types (Fig. 5 ▸).

For each protein, Asp and Asn exhibited qualitatively different behaviour, with the extended right-hand tail of the Asp distribution indicative of radiation-induced carboxyl­ate loss, in contrast to the radiation-insensitive amine functional group of Asn (Figs. 4*b*, 4*d*, 4*f*, 4*h* ▸). Conversely, Phe and Tyr exhibited qualitatively indistinguishable *D*_loss_ signatures (Figs. 4*a*, 4*c*, 4*e*, 4*g* ▸). Ile and Leu also showed qualitatively indistinguishable *D*_loss_ behaviour (Fig. S1.3), as expected, since both have similar aliphatic side-chain topologies that are believed to be relatively radiation-insensitive in protein crystals at 100 K, even at doses of several MGy.

As can be seen in Fig. 5 ▸, the Tyr–Phe KS-test statistic was consistently low and of the same order as the Leu–Ile control across the range of investigated proteins. The radiation effects to Phe and Tyr were thus deemed to be highly similar in protein crystals at 100 K in the MGy dose regime, with comparable spatial displacement of the aromatic rings. It is also noted that Thr and Ser exhibit comparable *D*_loss_ distributions, indicating that the presence of an additional methyl in Thr does not affect the damage to these groups.

**Figure 6:**
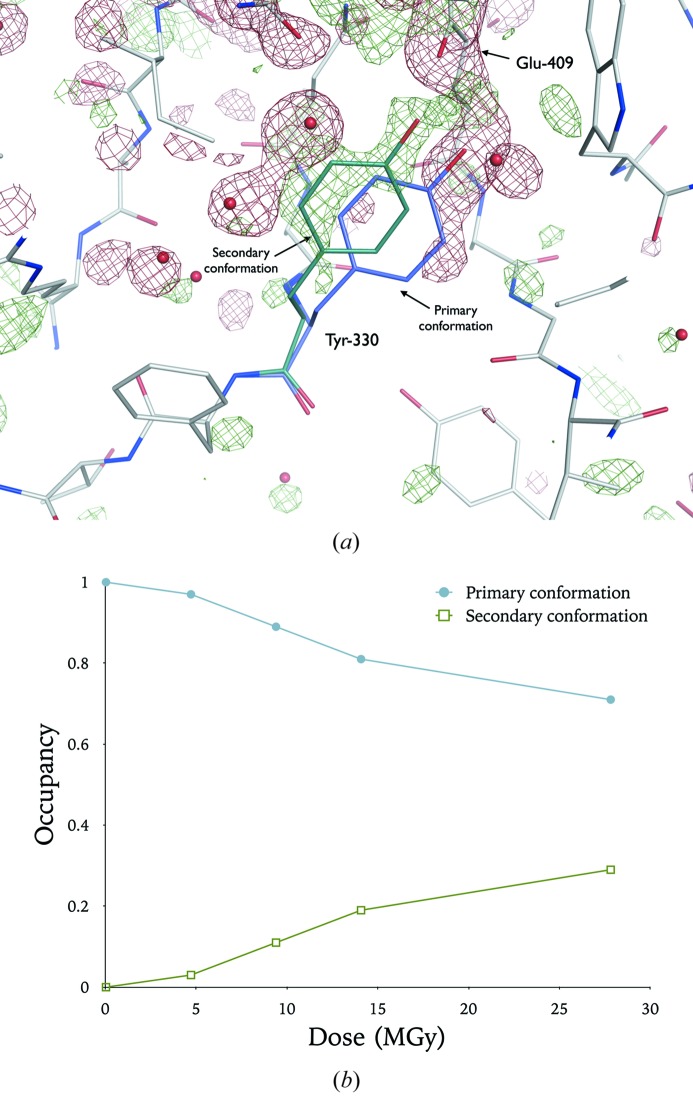
(*a*) Specific radiation damage to the myrosinase crystal as reported by Burmeister (2000 ▸). *F*_obs_(4) − *F*_obs_(1) difference map, contoured at ±3σ, overlaid on the initial coordinate model (PDB accession code: 1dwa). A secondary Tyr-330 side-chain conformation (occupancy: 50%) has been built into the positive *F*_obs_(4) − *F*_obs_(1) difference density manually using *COOT*. (*b*) Occupancy of primary and secondary Tyr-330 conformation after occupancy refinement (in *phenix.refine*, with atomic *B*-factors held constant) of the model pertaining to each successive dataset in the damage series. Doses were originally quoted in units of photons mm^−2^; diffraction weighted doses (DWD) (Zeldin, Brockhauser *et al.*, 2013 ▸) have been calculated in *RADDOSE-3D* (Zeldin, Gerstel *et al.*, 2013 ▸) for this damage series using crystal composition (heavy atom content, crystal size) and beam characteristics (energy, flux, exposure time and collimation) as supplied by Burmeister (2000 ▸).

To confirm whether the Tyr –OH density loss observations in myrosinase were consistent with full aromatic ring displacement, a secondary Tyr-330 aromatic ring conformation (initially with 50% occupancy) was built into the 1dwa coordinate model. With increasing dose, there is an observed shift in the relative occupancies of the two Tyr-330 conformations (Fig. 6 ▸). The growth in secondary conformation occupancy is consistent with partial reorientation of the Tyr-330 ring with dose. However, interestingly, despite the clear presence of a secondary conformation within the *F*_obs_(4) − *F*_obs_(1) map at a low σ-level (Fig. 6*a* ▸), for all doses within the myrosinase damage series, the formation of an unmodelled alternative Tyr-330 conformation was not detected using *RINGER* at low *2mF*_obs_ − *DF*_calc_ map σ-thresholds (above a default +0.3σ cutoff).

### 3.4. Factors affecting the Tyr –OH group   

**Figure 7:**
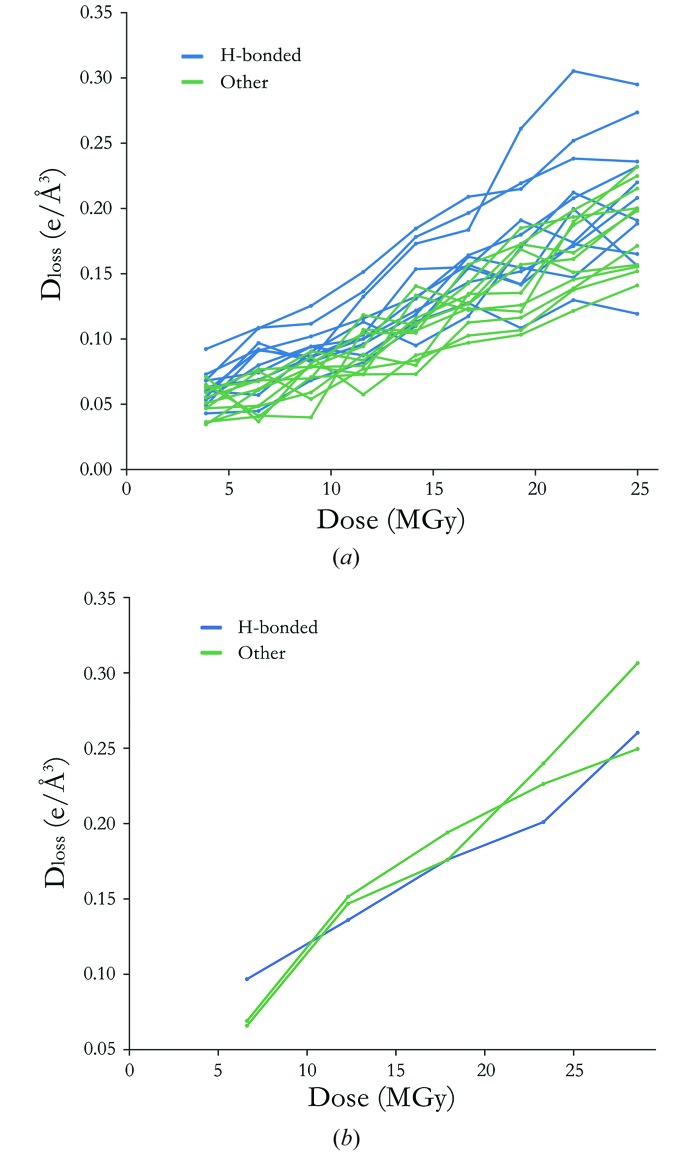
*D*_loss_(atom) metric as a function of dose for Tyr –OH atoms in (*a*) TRAP and (*b*) lysozyme (De la Mora *et al.*, 2011 ▸). Tyr –OH residues exhibiting hydrogen bond interactions to Glu or Asp carboxyl groups are coloured blue. Plots for other tested protein series are provided in Fig. S1.4.

To examine the dependence of Tyr –OH *D*_loss_ on availability of proximal hydrogen bond interactions to acidic residues, for each structure, Tyr residues were grouped into two subsets: those predicted to interact with Glu/Asp carboxyl­ates at distances under 4 Å (between the Tyr –OH oxygen and the carboxylate oxygens), and all other Tyr residues. Fig. 7 ▸ illustrates the results for two selected proteins, with the other systems presented in Fig. S1.4. Seven damage series contained a distinct subset of Tyr –OH atoms with *D*_loss_ exceeding that attained by a group of non-hydrogen bond-forming residues, whereas for six series no difference was observed between the two Tyr subgroups. In one protein [elastase (Petrova *et al.*, 2010 ▸)], no Tyr residues were predicted to form such hydrogen bonds. Only for two structures were the lowest *D*_loss_ values assigned to a subset of hydrogen bonded Tyr –OH groups [lysozyme (De La Mora *et al.*, 2011 ▸) and insulin (Nanao *et al.*, 2005 ▸)]. However, these were sampled over three and four Tyr groups, respectively, and are thus not considered statistically significant. Consequently, whereas Tyr –OH hydrogen bonding interactions with proximal Glu/Asp residues promoted radiation-induced Tyr –OH electron density loss in a subset of tested proteins (in strong support of prior anecdotal evidence), this was not uniformly reported for all proteins. Moreover, only for myrosinase (Burmeister, 2000 ▸) and thermolysin (Juers & Weik, 2011 ▸) were the collective group of hydrogen bond-forming Tyr –OH atoms statistically distinguishable from the remaining Tyr –OH groups (at α = 5% significance, Hotelling T^2^ test).

**Figure 8:**
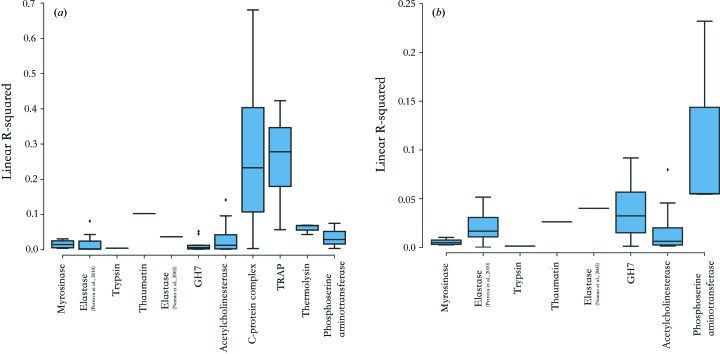
Linear *R*-squared coefficients calculated for each MX damage series for correlation between Tyr –OH *D*_loss_ and (*a*) solvent accessibility and (*b*) distance to closest di­sulfide bond. Box plots represent distribution in *R*-squared values for series containing multiple datasets. Only structures containing eight or more Tyr residues per asymmetric unit are included, and in (*b*) only structures containing di­sulfide bonds are included.

The loss of a direct Tyr–Glu/Asp hydrogen bond may not in itself be sufficient for increased Tyr ring flexibility, and undoubtedly other factors will influence the Tyr local environment: the identity of the remaining radicalized Glu/Asp species post de­carboxyl­ation, the proximity of additional hydrogen bond acceptors, and aromatic ring stacking interactions. Currently the relative importance of these factors is challenging to deconvolute and quantitate. To parametrize the reduction in Tyr steric hindrance following Glu/Asp de­carboxyl­ation, the solvent accessible area for each Tyr aromatic ring was calculated, after first removing all Glu and Asp carboxyl­ates from each protein coordinate model. Poor correlation was observed between Tyr –OH *D*_loss_ and the resulting solvent accessibility for each damage series (Fig. 8*a* ▸). It has been suggested that di­sulfide bond cleavage may induce changes to local protein backbone conformation and Tyr side-chain orientation, but, for the subset of di­sulfide containing proteins (Fig. 8*b* ▸), no correlation was detected between the Tyr –OH *D*_loss_ metric and the distance (Å) to di­sulfide bonds.

## 4. Discussion   

The characterization of radiation-mediated cleavage events in protein crystals remains an essential component of structure solution by crystallographic methods, with undetected specific damage currently being particularly detrimental to the success of modern time-resolved investigations aiming to unravel protein kinetics. The correct interpretation of electron density around each Tyr –OH moiety is often vital to ensure valid descriptions of the intramolecular interactions underpinning protein function. Tyrosine can play a characteristic role in signal transduction pathways, in which signalling specificity is dependent on direct enzyme-mediated group substitution (acyl­ation, phospho­rylation, sulfation) of the –OH functional group. In metalloproteins, tyrosine has also frequently been reported in the coordination sphere of the bound metal, with the Tyr phenolic oxygen directly acting as a donor to the metal cofactor.

The large body of radiation chemistry studies on both aqueous and solid state protein samples has been consulted to suggest feasible mechanisms for tyrosine-related damage events to crystalline proteins held at 100 K. Low-energy solvated electrons (∼1–10 eV) and positive holes are thought to be the dominant reactive species able to migrate at 100 K (Jones *et al.*, 1987 ▸), with less reactive hydrogen atoms also predicted to be mobile but produced in far lower yield than electrons upon water radiolysis (Spinks & Woods, 1990 ▸). The hydroxyl radical, known to be highly reactive from room-temperature protein and nucleic acid aqueous studies, is thought to be immobilized within the bulk solvent constituents of the crystal at 100 K (Owen *et al.*, 2012 ▸). However, for Tyr –OH removal, no such electron- or hole-driven mechanisms have been presented in the radiation chemistry literature, and the tyrosyl radical produced following deprotonation of the oxidized residue is deemed to form a stable redox couple with tyrosine. Indeed, in a neutral di­sulfide, the typical S—S bond strength is ∼55 kcal mol^−1^ (Benson, 1978 ▸). However, in the reduced species (the radical anion) that strength is lowered to ∼23 kcal mol^−1^ [derived from our quantum chemical calculations, as performed by Bury *et al.* (2016 ▸)]. By way of contrast in a phenol (such as tyrosine), we calculate that the C—O bond strength is approximately 110 kcal mol^−1^ and it is not significantly different in the oxidized species.

Re-investigation of the original myrosinase MX damage series data independently reproduced the observations of Tyr-330 –OH group electron density loss in *F*_obs_(*n*) − *F*_obs_(1) difference maps. In the Burmeister damage study, rigid body and overall anisotropic *B*-factor refinement (but no positional refinement) was performed starting from a 1.2 Å myrosinase model derived from a different crystal used for another experiment (Burmeister *et al.*, 2000 ▸). We suggest that this refinement strategy may account for the low density map-model RSCC calculated for the original data (0.67). However, whereas full re-refinement and model re-building with *PDB_REDO* resulted in a significant increase in RSCC, negative difference peaks surrounding the Tyr-330 –OH group were still detectable above noise in *F*_obs_(*n*) − *F*_obs_(1) maps. *PDB_REDO* re-refinement also provided an improved fit to *2mF*_obs_ − *DF*_calc_ map density for several Tyr aromatic rings (*e.g.* Tyr-215), and a pronounced negative difference density (comparable with that at Tyr-330) became interpretable around the Tyr-215 –OH group; a finding not originally reported by Burmeister (2000 ▸). Tyr-215 and Tyr-330 occupy similar hydrated pockets in myrosinase, with both being involved in hydrogen bond interactions (to Glu-153 and Glu-409, respectively), and both being within 5 Å of an ordered glycerol molecule. As such, the similar –OH electron density loss for Tyr-215 and Tyr-330 is perhaps not surprising.

The *D*_loss_ per-atom quantification of radiation-induced electron density loss has provided an invaluable tool to permit objective investigation of Tyr –OH density loss events in further protein damage series collected at 100 K. The available MX damage series deposited in the PDB gave a wide range of independently refined coordinate models corresponding to proteins of varying size (5.7–204 kDa), each with unique nuances of buffer composition (potential free radical scavengers) and macromolecular composition (monomeric or multimeric protein, presence of nucleic acid, glycans and metal cofactors). No restriction on resolution or crystallization cocktail composition has been enforced in the current sampling, and, accordingly, no attempt has been made to quantitatively compare raw *D*_loss_ values between proteins. However, as a testament to the strength of the *D*_loss_ metric, clear trends have emerged, with radiation-induced Glu and Asp de­carboxyl­ation highlighted to be prevalent across the range of protein systems in our study at doses >1 MGy. Prominent Tyr –OH density loss did not appear to be a widespread feature of general protein structures, a finding that is consistent with the high energetic barrier for Tyr phenolic C—O bond scission. It is thus arguable whether tyrosine should be included with Cys, Glu, Met and Asp as a radiation-sensitive locus.

The evidence suggests that (*a*) the Tyr and Phe aromatic rings undergo comparable positional displacement with increasing dose, and (*b*) Tyr –OH electron density loss is correlated with adjacent Tyr-C_ζ_ density loss [for structures in which high Tyr –OH *D*_loss_(atom) values have been reported here: myrosinase and TRAP with linear *R* ^2^ at 0.72 and 0.58 for the respective highest dose datasets, see §3 of the supporting information]. Overall these observations support a model of full aromatic ring displacement without the necessity of bond cleavage. Indeed, through occupancy refinement of multiple Tyr side-chain conformations, the original Burmeister (2000 ▸) results (Tyr-330) have been shown to be highly consistent with this postulate, since as the occupancy of the original conformation decreases, the population of alternative positions increases (Fig. 6*b* ▸).

There has been anecdotal evidence to suggest a relationship between radiation-induced Tyr –OH electron density dis­ordering and the presence of hydrogen bond interactions to Glu/Asp carboxyl side-groups. Indeed, in TRAP (Bury *et al.*, 2016 ▸), a macromolecule with 11-fold rotational self-symmetry, 11 symmetrically related Tyr copies all hydrogen bond to nearby Glu residues, and all exhibit *F*_obs_(*n*) − *F*_obs_(1) map peaks that are distinguishable above noise.

In the current study, whereas select Tyr residues forming hydrogen bond interactions to proximal Glu/Asp carboxyl­ates exhibited comparably high *D*_loss_ metric values (relative to non hydrogen bond-forming Tyr –OH groups), this behaviour was restricted to a subset of proteins (myrosinase, TRAP, thermolysin, insulin), and was not conserved amongst all such Tyr residues for these proteins. We suggest that a multitude of compounding factors associated with a hydrogen bonding interaction must play a role in the density loss rate for each individual Tyr –OH group (*e.g.* readiness of Glu/Asp de­carboxyl­ation, lability of the Tyr aromatic group, steric hindrance both prior and post de­carboxyl­ation). However, no quantitative correlation could be established for these individual factors. For example, considering the two most susceptible Tyr –OH groups in myrosinase (Burmeister, 2000 ▸), following loss of interacting Glu –CO_2_ moieties, a negligible change in overall aromatic ring solvent accessible area (used as a measure for steric hindrance) was detected for Tyr-330, whereas an increase of 1.36 Å^2^ was observed for Tyr-215.

Nonetheless, the top three Tyr residues in myrosinase flagged by the *Bdamage* metric (Tyr-215, Tyr-330, Tyr-390) do all exhibit hydrogen bonding interactions to glutamates. We suggest that the high relative *Bdamage* change values for the active site residues Tyr-215 and Tyr-330 are a direct indication of relaxation of the strained active site with accumulated dose. This finding is in close accord with the consensus that active sites are particularly prone to specific damage events (Holton, 2009 ▸; Dubnovitsky *et al.*, 2005 ▸). For the full set of myrosinase tyrosine –OH oxygen atoms, a strong positive correlation was found between *D*_loss_ and change in *Bdamage* between the first and higher dose datasets (Fig. S1.5, linear *R* ^2^ > 0.7 for all higher dose datasets). Overall, the *potential flexibility* of each Tyr appears to directly correlate with electron density loss around the –OH group. The radiation-induced conformational shift for Tyr-330 is pronounced since this residue is purposefully labile in order to reorientate to accept glucose into the binding pocket. However, such a tyrosine conformational shift is an indirect effect of nearby radiation chemistry (*e.g. *disulfide bond cleavage, de­carboxyl­ation), and thus should not be considered a feature exclusive to tyrosines.

## 5. Related literature   

The following references, not cited in the main body of the paper, have been cited in the supporting information: Chen *et al.* (2010 ▸); Fuentes-Montero *et al.* (2014 ▸); McCoy *et al.* (2007 ▸); Southworth-Davies *et al.* (2007 ▸).

## Supplementary Material

Suppoorting information. DOI: 10.1107/S1600577516016775/xh5049sup1.pdf

## Figures and Tables

**Table 1:** Selection of MX damage series (all conducted at 100 K) previously deposited in the PDB, complemented with one novel GH7 family protein damage series (deposited in conjunction with this study) and one unpublished insulin data set. See §2 of the supporting information for data collection and refinement of the GH7 series For series in which intermediate datasets fall below the 1 MGy selection criterion, PDB accession codes are only provided for datasets included in the current analysis.

Protein	PDB accession codes	Resolution for first dataset (Å)	Quoted total molecular weight (kDa)	Unique Tyr residues in asymmetrical unit	Publication
Acetyl­cholinesterase (*Torpedo californica*)	1qid, 1qie, 1qif, 1qig, 1qih, 1qii, 1qij, 1qik, 1qim	2.05	60.7	17	Weik *et al.* (2000 ▸)
Thermolysin (*Bacillus thermoproteolyticus*)	3p7p, 3p7q, 3p7r, 3p7s, 3p7t, 3p7u, 3p7v, 3p7w	2.2	34.6	28	Juers & Weik (2011 ▸)
Myrosinase (*Sinapis alba)*	1dwa, 1dwf, 1dwg, 1dwh	2.0	62.4	37	Burmeister (2000 ▸)
Malate de­hydrogenase (*Haloarcula marismortui*)	2j5k, 2j5q, 2j5r	2.0	131.6	32	Fioravanti *et al.* (2007 ▸)
Chicken egg white lysozyme (*Gallus gallus*)	2ybh, 2ybi, 2ybj, 2ybl, 2ybm, 2ybn	2.0	14.6	3	De la Mora *et al.* (2011 ▸)
Chicken egg white lysozyme (*Gallus gallus*)	4h8x, 4h8y, 4h8z, 4h90, 4h91, 4h92, 4h93, 4h94, 4h9a, 4h9b, 4h9c, 4h9e, 4h9f, 4h9h, 4h9i	1.2	14.5	3	Sutton *et al.* (2013 ▸)
Pancreatic elastase (*Sus scrofa)*	3mnb, 3mnc, 3mns, 3mnx, 3mo3, 3mo6, 3mo9, 3moc	1.2	26.1	11	Petrova *et al.* (2010 ▸)
Pancreatic elastase (*Sus scrofa)*	2blo, 2blq	1.33	26.2	11	Nanao *et al.* (2005 ▸)
Insulin (*Bos taurus)*	2bn3, 2bn1	1.4	5.8	4	Nanao *et al.* (2005 ▸)
Lysozyme (*Gallus gallus)*	2blx, 2bly	1.4	14.7	3	Nanao *et al.* (2005 ▸)
Ribonuclease A (*Bos taurus)*	2blp, 2blz	1.4	13.7	6	Nanao *et al.* (2005 ▸)
Thaumatin (*Thaumatococcus daniellii)*	2blr, 2blu	1.4	22.3	8	Nanao *et al.* (2005 ▸)
Trypsin (*Bos taurus)*	2blv, 2blw	1.2	24.0	10	Nanao *et al.* (2005 ▸)
C-protein-DNA complex (*Escherichia coli*)	4x4b, 4x4c, 4x4d, 4x4e, 4x4f, 4x4g, 4x4h, 4x4i	2.8	59.7	8	Bury *et al.* (2015 ▸)
*Trp* RNA-binding attenuation protein (TRAP) -RNA complex (*Escherichia coli*)	5eeu, 5eev, 5eew, 5eex, 5eey, 5eez, 5ef0, 5ef1, 5ef2, 5ef3	1.98	204.1	22	Bury *et al.* (2016 ▸)
Phospho­serine amino­transferase *(Bacillus alcalophilus)*	2bhx, 2bi5, 2bi9, 2bia	1.68	81.8	28	Dubnovitsky *et al.* (2005 ▸)
Insulin (*Bos taurus)*	Undeposited damage series	1.38	5.8	4	Current publication
GH7 family cellobio­hydro­lase *(Daphnia pulex)*	5mcc, 5mcd, 5mce, 5mcf, 5mch, 5mci, 5mcj, 5mck, 5mcl, 5mcm, 5mcn	2.0	96.6	34	Unpublished
