# Supplementary material for: OH cleavage from tyrosine: debunking a myth
Source: J Synchrotron Radiat. 2017 Jan 1;24(Pt 1):7–18. doi: 10.1107/S1600577516016775 (PMC5182017; doi:10.1107/S1600577516016775)
Supplement: Supplementary file 1 [file s-24-00007-sup1.pdf]

JOURNAL OF SYNCHROTRON RADIATION

Volume 24 (2017)

Supporting information for article:

OH cleavage from tyrosine: debunking a myth

Charles S. Bury, Ian Carmichael and Elspeth F Garman

OH cleavage from tyrosine: debunking a myth

Charles S. Bury[a], Ian Carmichael[b], Elspeth F. Garman[a*]

[a] Laboratory of Molecular Biophysics, Department of Biochemistry, University of Oxford, South Parks Road, Oxford, OX1 3QU, UK

[b] Notre Dame Radiation Laboratory, University of Notre Dame, Notre Dame, IN 46556, USA

* corresponding author elspeth.garman@bioch.ox.ac.uk

Supplementary material 1: Figures and Tables

Table S1.1. R_{work} and R_{free} statistics corresponding to the lowest dose structures for each MX damage series located within the PDB, as reported in original coordinate file header retrieved from the PDB, in addition to values obtained following *REFMAC* rigid body refinement cycles in *PDB_REDO (*either 0 or 10 rounds depending on whether potential R_{free} bias was flagged by *PDB_REDO)*. The percentage difference in R_{work} and R_{free} between the two sources is also provided, as are any warning flags raised by *PDB_REDO* during data handling. For all structures in which no possible R_{free} bias flag was raised, both R_{work} and R_{free} recalculated by *PDB_REDO* following rigid body refinement agreed with the original values reported in the PDB to within 2%. Myrosinase was the only structure reported here for which both the recalculated R_{work} and R_{free} (after 10 cycles of rigid body refinement through *PDB_REDO*) exceeded the original PDB header values by 2%.

Publication	Protein	PDB code	Original PDB R_{work}/R_{free}	*PDB_REDO* (rigid body refinement): R_{work}/R_{free}	% difference R_{work}/R_{free}	*PDB_REDO* Notes
Weik *et al.,* 2000	Acetylcholinesterase	1qid	0.22/0.23	0.21/0.21	-0.49/-1.84	Possible R_{free} bias flagged
Juers & Weik, 2011	Thermolysin	3p7p	0.15/0.21	0.15/0.20	0.15/-0.76	-
Burmeister, 2000	Myrosinase	1dwa	0.17/0.18	0.19/0.20	2.6/2.23	Possible R_{free} bias flagged
Fioravanti *et al.,* 2007	Malate dehydrogenase	2j5k	0.23/0.27	0.22/0.22	-0.09/-4.86	New R_{free} set (5%)
De la Mora *et al.,* 2011	Lysozyme	2ybh	0.20/0.24	0.20/0.21	-0.32/-3.07	New R_{free} set (10%)
Sutton *et al.,* 2013	Lysozyme	4h8x	0.19/0.20	0.18/0.19	-0.76/-0.82	-
Petrova *et al.,* 2010	Elastase	3mnb	0.11/0.14	0.11/0.14	0.07/-0.09	-
Nanao *et al.,* 2005	Elastase	2blo	0.11/0.14	0.11/0.14	0.06/-0.04	-
Nanao *et al.,* 2005	Insulin	2bn3	0.13/0.16	0.13/0.16	-0.05/-0.09	-
Nanao *et al.,* 2005	Lysozyme	2blx	0.13/0.17	0.13/0.17	0.09/-0.11	-
Nanao *et al.,* 2005	Ribonuclease A	2blp	0.15/0.18	0.15/0.17	0.09/-0.24	-
Nanao *et al.,* 2005	Thaumatin	2blr	0.13/0.16	0.12/0.15	-0.39/-0.22	-
Nanao *et al.,* 2005	Trypsin	2blv	0.11/0.13	0.11/0.13	0.09/0.23	-
Bury *et al.,* 2015	C-protein DNA	4x4b	0.21/0.26	0.22/0.27	1.85/0.89	-
Bury *et al.,* 2016	Trp RNA-binding Attenuation Protein	5eeu	0.19/0.23	0.19/0.23	-0.01/-0.28	-
Dubnovitsky *et al.,* 2005	Phosphoserine aminotransferase	2bhx	0.18/0.23	0.17/0.18	-0.97/-5.07	Possible R_{free} bias flagged

Table S1.2. Distribution of real-space Z_{obs} *(RSZO)* scores calculated by *EDSTATS* (Tickle, 2012) for the *1dwa* structure retrieved directly from PDB and retrieved from *PDB_REDO (Joosten et al., 2014)* after 10 cycles of rigid body refinement. *RSZO* is a metric for model precision and is calculated as $RSZO = mean(\rho_{obs}) / \sigma(\Delta\rho)$, in which $\sigma(\Delta\rho)$ is the standard uncertainty of the *1dwa* F_{obs}-F_{calc} map. The percentage of residues within *1dwa* with lower *RSZO* scores than specific thresholds (between 0.5 and 3) are provided. The *RSZO* score is unbounded from above, with larger positive values indicating greater residue to density fit (Tickle (2012)). For an individual residue, *EDSTATS* recommends a default <1 rejection threshold. For the *1dwa* coordinate model retrieved directly from the PDB, a significantly larger number of residues attain lower (poorer) *RSZO* scores, compare to the *1dwa* data retrieved from *PDB_REDO* instead.

	**Percentage of residues within myrosinase *1dwa* structure**					
	**Retrieved from PDB**			**Retrieved from PDB_REDO**		
**RSZO score**	*Main (%)*	*Side (%)*	*Overall (%)*	*Main (%)*	*Side (%)*	*Overall (%)*
0.5	0.2	3.0	2.8	0.0	0.0	0.0
1.0	3.0	18.6	16.6	0.2	1.8	1.8
1.5	22.2	42.3	42.3	0.8	6.4	6.0
2.0	75.8	80.2	90.4	1.4	13.8	12.2
2.5	99.2	96.1	99.8	3.4	19.1	17.0
3.0	100.0	97.5	100.0	6.2	24.8	22.4

Figure S1.1. $2mF_{obs}$ - DF_{calc} electron density maps and mF_{obs} - DF_{calc} maps for the *1dwa* myrosinase model at different stages in the *PDB_REDO* re-refinement, centred at residue Tyr-215. All $2mF_{obs}$ - DF_{calc} and mF_{obs} - DF_{calc} maps were generated over the asymmetric unit with *FFT,* and are contoured at 2 (in grey) and +/–4 σ (green/red) respectively. (a) The original *1dwa* model extracted directly from the PDB (with ordered water in red), with the corresponding original electron density maps (maps were extracted from the PDB in .mmcif format, and converted to .mtz format with CCP4 program *CIF2MTZ*). In (b), the coordinate model after 10-cycles of rigid body refinement with *PDB_REDO* has been superimposed on the coordinate model and electron density maps in (a). (c) The re-refined *1dwa* model (with ordered water in yellow) and electron density maps extracted from *PDB_REDO* after 10-cycles of rigid body refinement. In (d), the coordinate model after full model re-building with *PDB_REDO* has been superimposed on the coordinate model and electron density maps in (c). (e) The fully re-refined *1dwa* model (with ordered water in pink) and electron density maps extracted from *PDB_REDO* after full model re-building. Full model re-building with *PDB_REDO* resulted in correct placement of both the Tyr-215 aromatic group and the ordered solvent molecules. All subfigures were rendered in *PyMOL* (www.pymol.org).

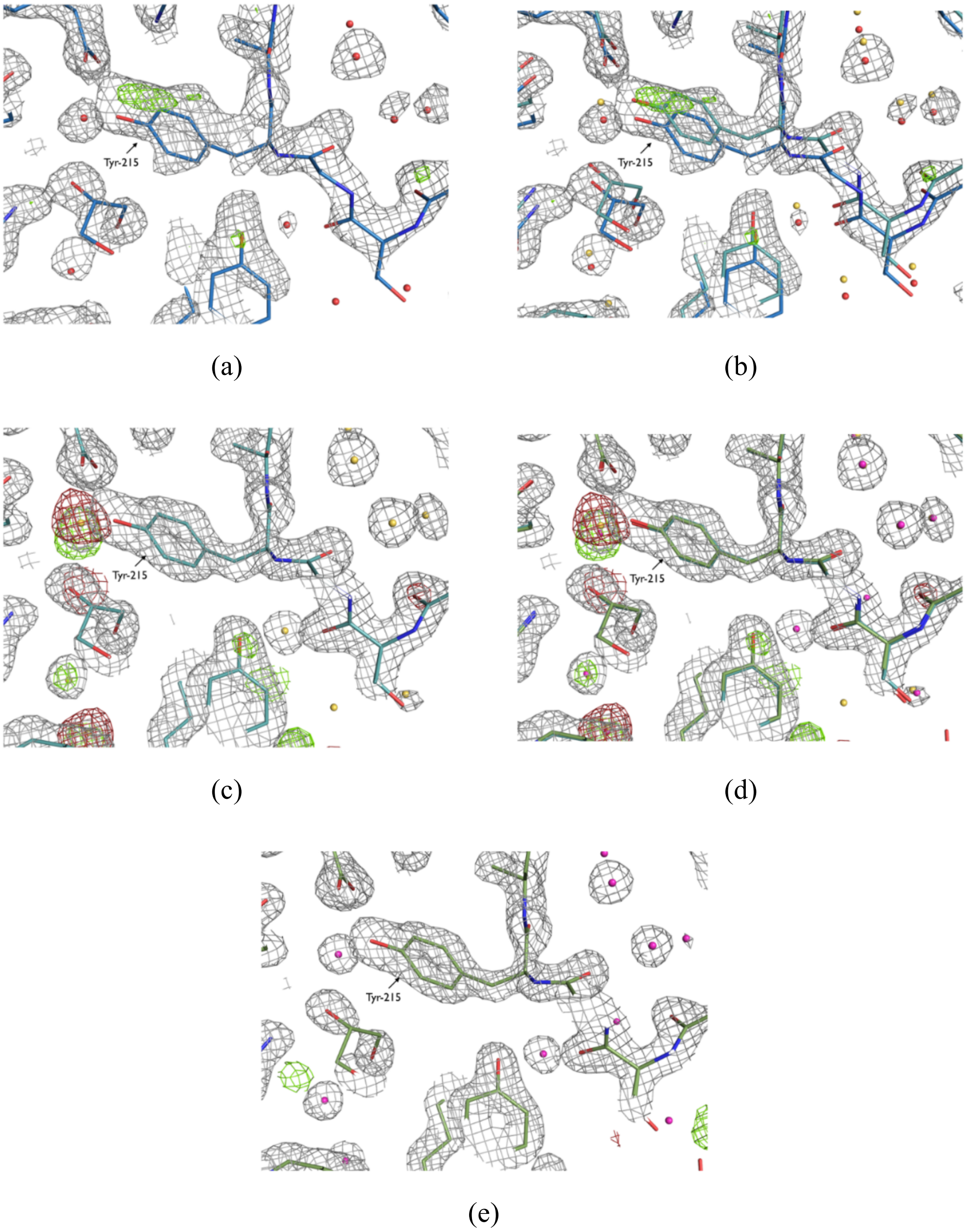

Figure S1.2. Specific radiation damage to Tyr-215 in the myrosinase damage series (Burmeister, 2000). (a) $F_{obs}(5)$ - $F_{obs}(1)$ difference map, overlaid on the original initial coordinate model in white (PDB accession code: *1dwa*). Structure factor amplitudes have been retrieved directly from the PDB for the $F_{obs}(5)$ - $F_{obs}(1)$ map calculation, with phases derived from the original 1dwa coordinate model. Negative difference density is observable adjacent to the Tyr-215, however does not align with the –OH group. (b) $F_{obs}(5)$ - $F_{obs}(1)$ difference map, overlaid on the re-refined initial coordinate model in white (PDB accession code: *1dwa*) retrieved from *PDB_REDO*. Structure factor amplitudes have been retrieved from *PDB_REDO for* the $F_{obs}(5)$ - $F_{obs}(1)$ map calculation, with phases derived from the re-refined *1dwa* coordinate model. Clear negative difference density is observable centred at the position of the Tyr-215 –OH group. In both cases, the $F_{obs}(5) – F_{obs}(1)$ maps have been generated by *FFT* within the *RIDL* pipeline, and are overlaid over the coordinate models at +/-5 σ contouring levels in green/red.

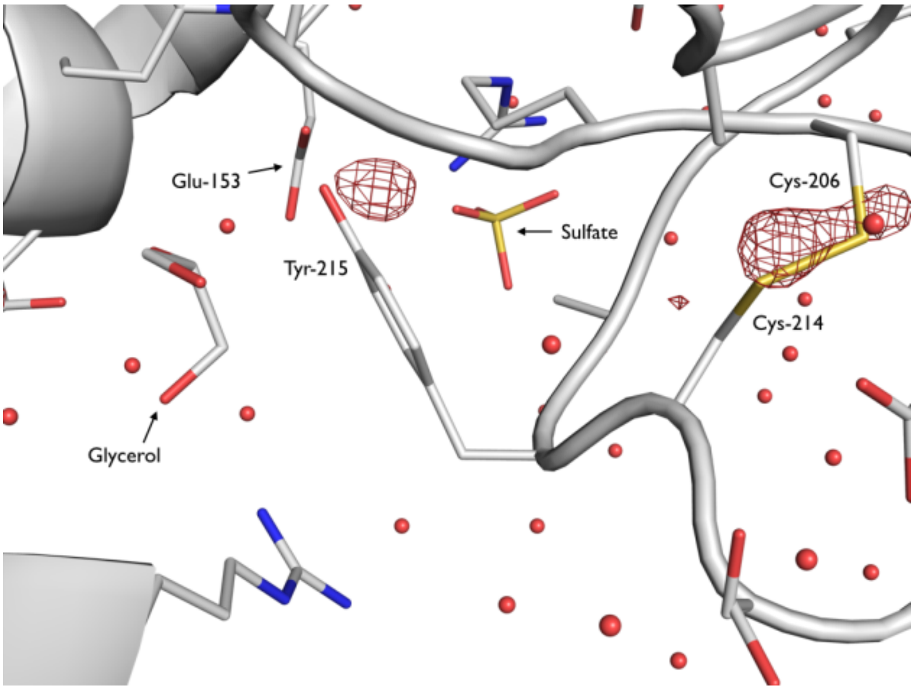

(a)

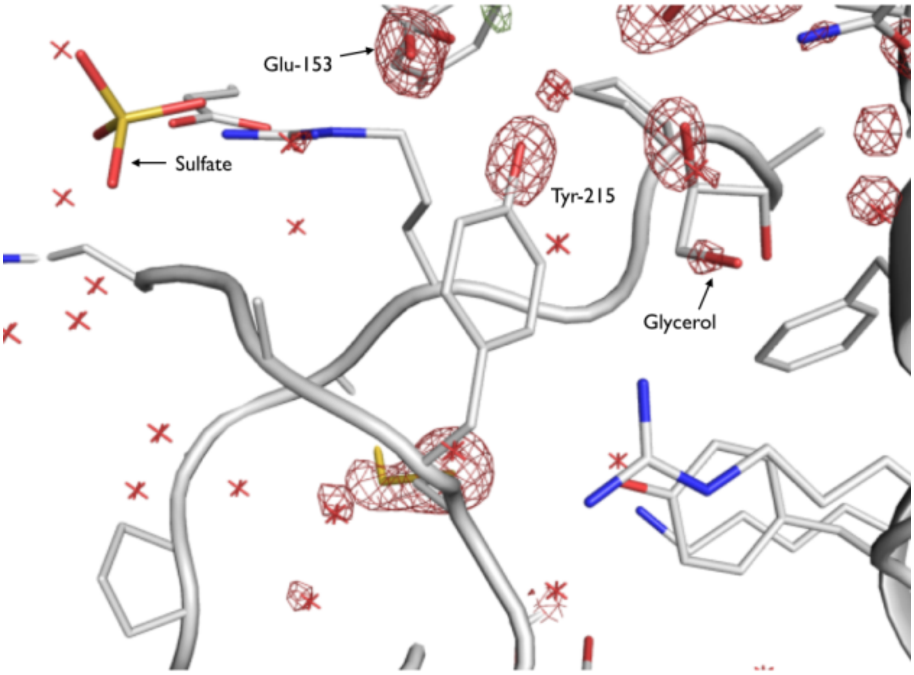

(b)

Figure S1.3. D_{loss} damage signature histogram plots over all atoms of selected residue types for (a,b) myrosinase (Burmeister, 2000), (c,d) TRAP (Bury *et al.*, 2016), and (e,f) Malate Dehydrogenase (Fioravanti *et al.*, 2007). Gaussian kernel density estimates are overlaid on the histogram plots. For each plot, Kolmogorov-Smirnov (KS) test statistics have been calculated to measure the similarity between the two residue types included. Other doses within these damage series exhibit qualitatively similar behavior (data not shown).

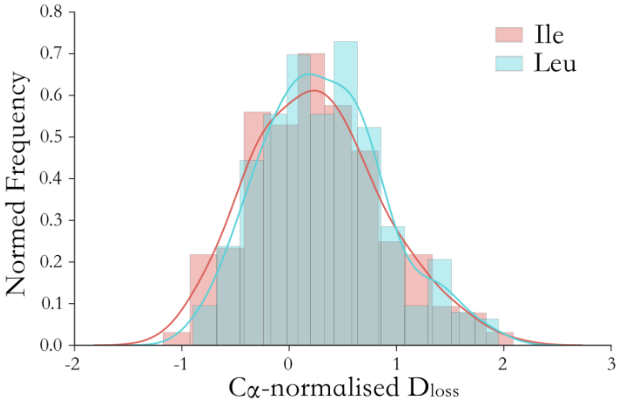

(a) KS stat: 0.089

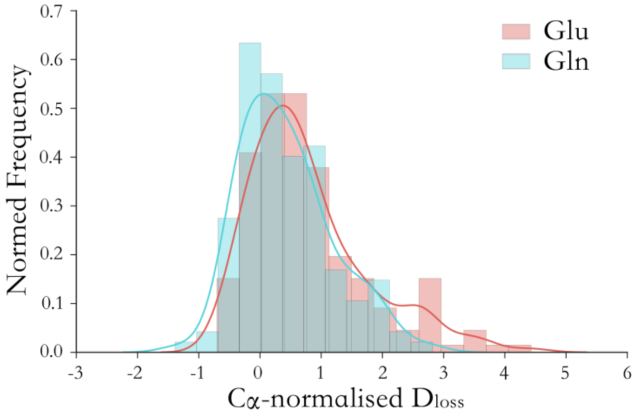

(b) KS stat: 0.181

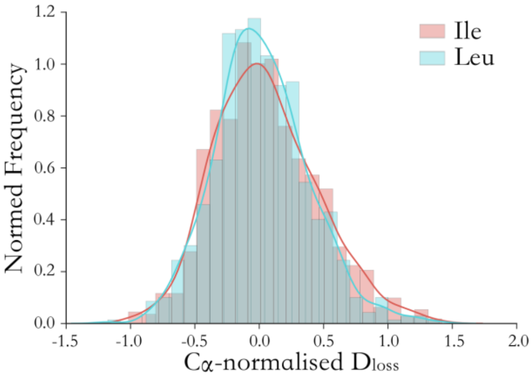

(c) KS stat: 0.059

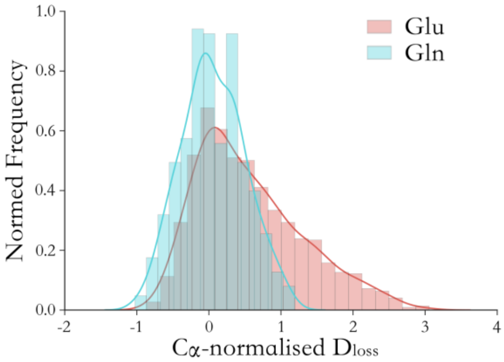

(d) KS stat: 0.306

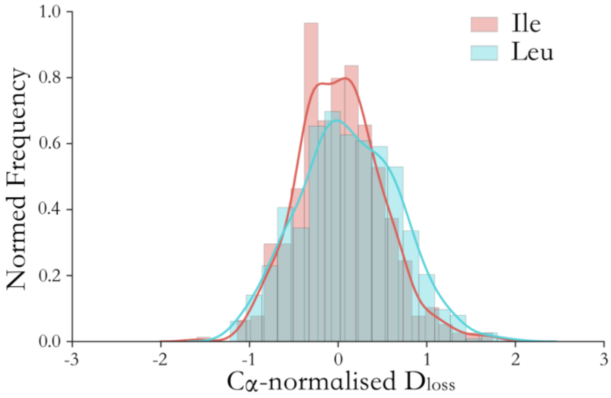

(e) KS stat: 0.13

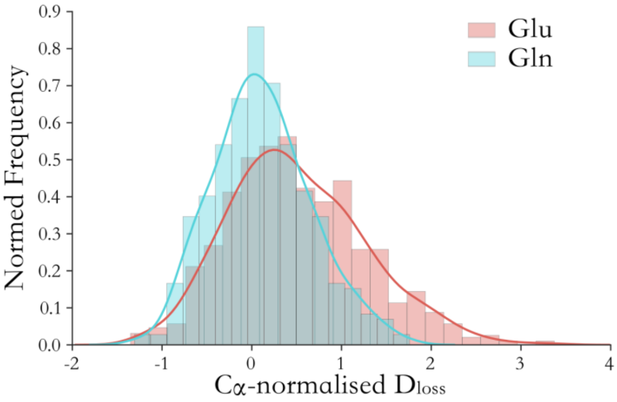

(f) KS stat: 0.248

Figure S1.4. D_{loss}(atom) metric as a function of dose for Tyr-OH atoms in (a) myrosinase, (b) thermolysin, (c) GH7, (d) TRAP, and (e) lysozyme (De La Mora *et al.*, 2011), (f) insulin (unpublished data), (g) the C-protein DNA complex, (h) phosphoserine aminotransferase, and (i) acetylcholinesterase. Tyr-OH residues exhibiting hydrogen bond interactions to Glu or Asp carboxyl groups are coloured blue. See main text for list of original publications for each protein structure. For clarity, only proteins for which damage series consisted of > 2 higher dose datasets have been included. *X*-axis doses are those reported originally (see original publications for corresponding dose calculations). The exception is myrosinase, for which doses were originally quoted in units of photons mm^{-2}; diffraction weighted doses (DWD) (Zeldin, Brockhauser*, et al.*, 2013) have been calculated in RADDOSE-3D (Zeldin, Gerstel*, et al.*, 2013) for this damage series using crystal composition (heavy atom content, crystal size) and beam characteristics (energy, flux, exposure time, and collimation) as supplied in (Burmeister, 2000).

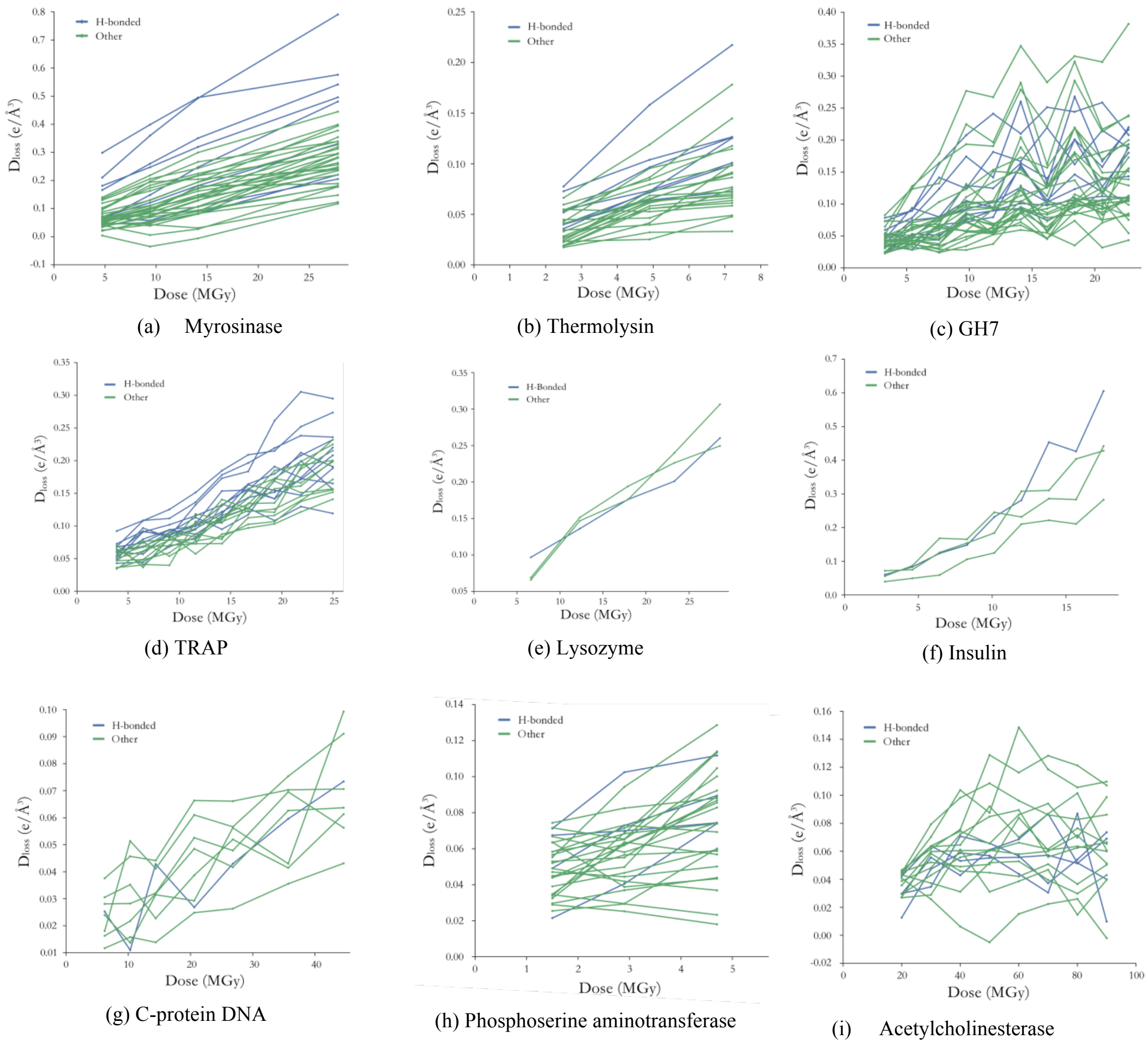

Figure S1.5. Relationship between D_{loss}(atom) and change in *Bdamage* (Gerstel *et al.*, 2015) relative to the *1dwa* structure, for each Tyr –OH atom in the myrosinase structure at each higher dose in series (a-d). For each atom *a*, and higher dose structure *k* = *1dwf, 1dwg, 1dwh, 1dwi,* the relative change is calculated as $[Bdamage(a^k) - Bdamage(a^{1dwa})]/Bdamage(a^{1dwa})$. To account for non-unity occupancies in the originally deposited data, all atomic occupancies in each coordinate model were set to 1 and a further round of isotropic *B*-factor refinement was performed in *phenix.refine (Adams et al., 2010)* prior to calculating *Bdamage*. The high correlation between D_{loss} and change in *Bdamage* is striking, since both are independently calculated radiation damage metrics. Whereas *Bdamage* is dependent on refined coordinate model atomic *B*-factor values, D_{loss} is derived directly from electron $F_{obs}(n) - F_{obs}(1)$ difference density values.

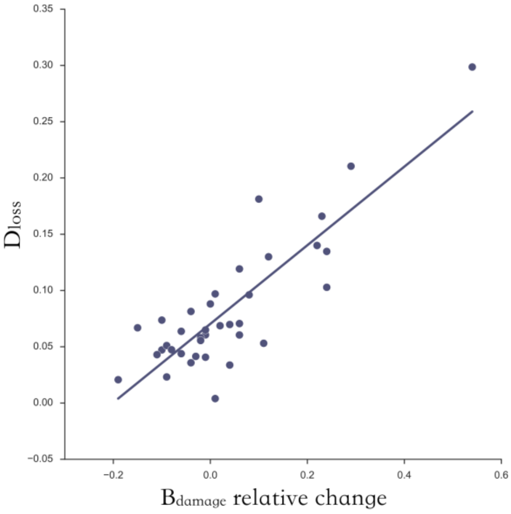

(a) R^2: 0.73

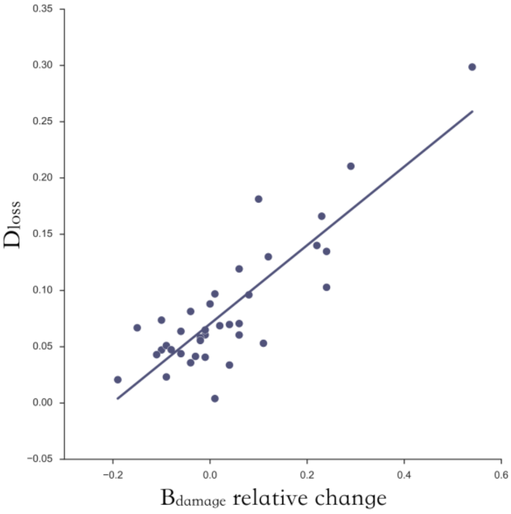

(b) R^2: 0.78

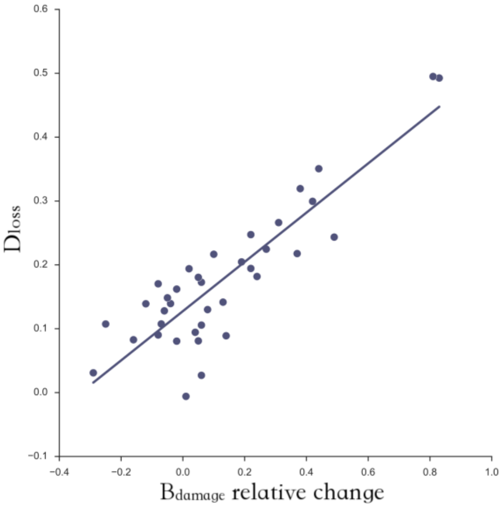

(c) R^2: 0.76

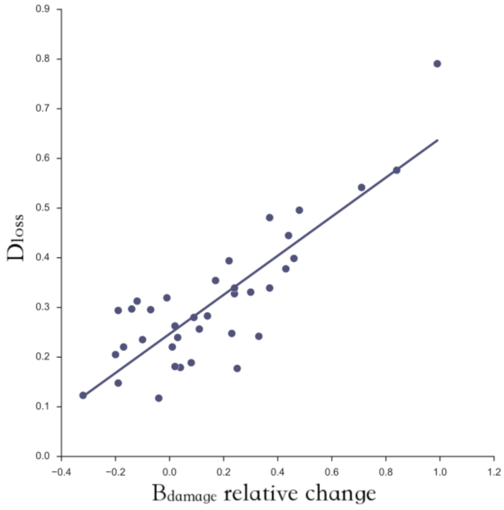

(d) R^2: 0.72

Supplementary 1 References

Adams, P. D., Afonine, P. V., Bunkóczi, G., Chen, V. B., Davis, I. W., Echols, N., Headd, J. J., Hung, L.-W., Kapral, G. J., Grosse-Kunstleve, R. W., McCoy, A. J., Moriarty, N. W., Oeffner, R., Read, R. J., Richardson, D. C., Richardson, J. S., Terwilliger, T. C. & Zwart, P. H. (2010). *Acta Cryst.* D**66**, 213-221.

Burmeister, W. P. (2000). *Acta Cryst.* D**56**, 328-341.

Bury, C. S., McGeehan, J. E., Antson, A. A., Carmichael, I., Gerstel, M., Shevtsov, M. B. & Garman, E. F. (2016). *Acta Cryst.* D**72**, 648-657.

De La Mora, E., Carmichael, I. & Garman, E. F. (2011). *J Synchrotron Rad.* **18**, 346-357.

Fioravanti, E., Vellieux, F. M., Amara, P., Madern, D. & Weik, M. (2007). *J Synchrotron Rad.* **14**, 84-91.

Gerstel, M., Deane, C. M. & Garman, E. F. (2015). *J Synchrotron Rad.* **22**, 201-212.

Joosten, R. P., Long, F., Murshudov, G. N. & Perrakis, A. (2014). *IUCrJ* **1**, 213-220.

Tickle, I. J. (2012). *Acta Cryst.* D**68**, 454-467.

Zeldin, O. B., Brockhauser, S., Bremridge, J., Holton, J. M. & Garman, E. F. (2013). *Proc. Natl. Acad. Sci. USA* **110**, 20551-20556.

Zeldin, O. B., Gerstel, M. & Garman, E. F. (2013). *J. Appl. Cryst.* **46**, 1225-1230.

Supplementary material 2: GH7 protein crystallization and data collection

Crystallization

Glycoside hydrolase family 7 cellulase from *Daphnia pulex* (DpCel7B) was supplied by the National Renewable Energy Laboratory (NREL), having been overexpressed in a *trichoderma reesei* system. A crystal was grown using a hanging-drop crystallization protocol with 2.75 mg/ml protein mixed with 0.1 M monosodium citrate and 0.9 M ammonium sulphate, pH 4. The approximately cuboid-shaped crystal had dimensions of 75 × 75 × 10 µm. Prior to cryocooling of the crystal, it was soaked for 2 minutes in buffer solution with 30% (v/v) glycerol added as a cryoprotectant, immediately before storage in liquid nitrogen.

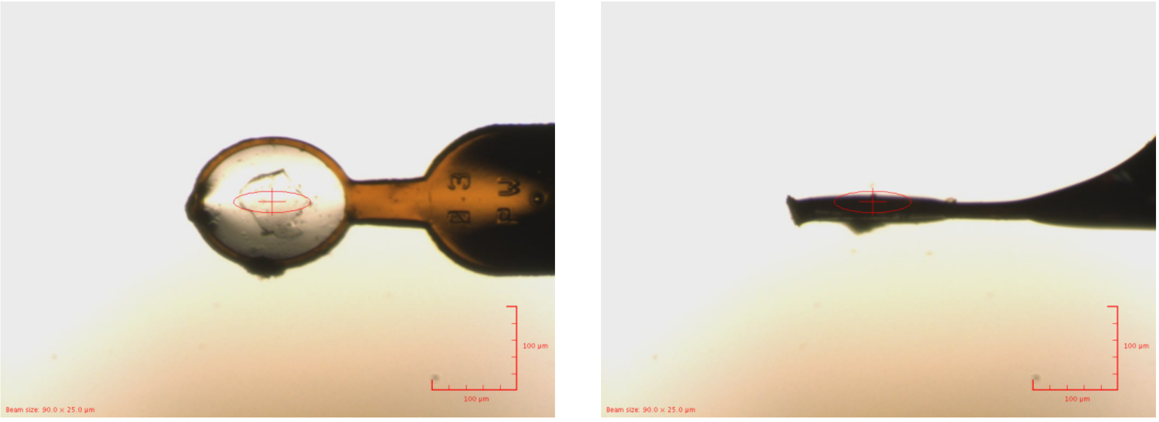

(a) (b)

Figure S2.1. A crystal of GH7 family protein in loop on beamline I02 at the Diamond Light Source. (a) Initial orientation of crystal with the smallest crystal dimension (z = 10 µm) parallel to the beam direction, and (b) 90° rotation of the loop in the beam path.

X-ray data collection

Data were collected at 100 K on beamline I02 at the Diamond Light Source, using an incident wavelength of 0.980 Å (12.7 keV) and a Pilatus 6M detector, positioned at 244.9 mm from the crystal throughout data collection. The beam passed through a 200 µm aperture and was slitted to 60 × 120 µm (vertical x horizontal), with an approximate Gaussian profile (vertical × horizontal FWHM: 17.7 × 104.8 µm). The flux at the sample position was determined using a pre-calibrated 500 µm thick silicon PIN photodiode to be ~1.77 × 10^{12} ph/s at 100% beam transmission. The crystal was initially orientated with the small crystal dimension (z = 10 µm) parallel to the beam direction. A 2000° continuous sweep of data was collected, consisting of 9999 frames ($\Delta\varphi$ = 0.2°) of 0.04 s exposure time each. The beam transmission was held at 25% throughout.

Data processing

The single of 2000° sweep of diffraction data was divided into 11 disjoint 180° wedges (sufficient for completeness due to the C121 space group), each consisting of 900 images. Each wedge of data was integrated using *DIALS* (Fuentes-Montero *et al.,* 2014), and then scaled and merged in *AIMLESS* (Winn *et al.*, 2011). To obtain an initial set of phases for the first dataset, molecular replacement was performed in *PHASER* (McCoy *et al.,* 2007), using an identical GH7 family Cellobiohydrolase from *Daphnia pulex* already deposited in the PDB (accession code: *4xnn*, resolution 1.9 Å, (McGeehan, in preparation)) as a search model. The resulting initial coordinate model was then refined using *REFMAC5* (Murshudov *et al.*, 2011), first with 10 cycles of rigid body refinement, followed by repeated rounds of 10 cycles of restrained and TLS refinement, coupled with manual inspection in *COOT*. Final protein geometry was assessed with *Molprobity4* (Chen *et al.*, 2010).

For the model from each later dataset, 10 cycles of rigid body refinement were performed in *REFMAC*, using the refined coordinate model derived from the initial dataset coupled with the merged structure factors from the later dataset. This is a standard protocol for refining multi-dataset protein damage series (Southworth-Davies *et al.*, 2007). Since the crystal unit cell dimensions generally increase as a function of dose (Ravelli & McSweeney, 2000), rigid body refinement was employed to compensate for slight re-definition of the unit cell parameters with increasing dose. For the current analysis, no restrained refinement was performed for the models from each of the higher dose datasets. The calculation of the D_{loss} metric is derived from $F_{obs}(n) - F_{obs}(1)$ Fourier difference map coefficients, using the phases obtained for the initial dataset refined coordinate model, and as such restrained refinement of models for higher dose datasets is not required. Data reduction and refinement statistics for the full damage series are reported in Tables S2.1 and S2.2. Coordinates and structure factor amplitudes have been deposited in the PDB with accession codes: *5mcc, 5mcd, 5mce, 5mcf, 5mch, 5mci, 5mcj, 5mck, 5mcl, 5mcm* and *5mcn*.

Dose calculations

The diffraction weighted dose (DWD) was calculated using RADDOSE-3D for each dataset in the damage series. The crystal was modelled as a cuboid with dimensions 75 × 75 × 10 µm. The crystal composition was determined using the *4xnn* coordinate model, which has an identical sequence. The contribution of heavy elements within the crystallization buffer (0.1 M Na and 0.9 M S) was included in the calculated crystal absorption coefficients. A Gaussian-shaped beam was modelled with parameters as given above. As a result of the high symmetry in the data collection procedure, for dataset $n = 1,2,\ldots$ the resulting DWD value was calculated to be $1.11 + 2.16\times(n - 1)$ MGy. By the end of the final dataset ($n = 11$) the crystal had absorbed an accumulated dose of DWD = 22.7 MGy.

Table S2.1: Data processing and refinement statistics for GH7 family protein damage series. Values in parentheses are for the highest-resolution shells (fixed at 2.05 – 2.00 Å throughout). For observed F_{obs} and calculated F_{calc} structure factors, $R_{work} = \sum \frac{|F_{obs}-F_{calc}|}{\sum F_{obs}}$ R_{free} is the R_{work} formula calculated from a small (5%) test set of randomly selected reflections (constant 5% test set maintained between datasets in series). Unit cell dimensions of α = γ = 90° are conserved throughout. PDB accession codes are provided for the deposited series.

Dataset	1	2	3	4	5	6	7	8	9	10	11
**PDB codes**	5mcc	5mcd	5mce	5mcf	5mch	5mci	5mcj	5mck	5mcl	5mcm	5mcn
**Dose (MGy)**	1.11	3.27	5.43	7.59	9.75	11.9	14.1	16.2	18.4	20.6	22.7
**Resolution range (Å)**	164.1 – 2.00	82.1 - 2.00	82.3 - 2.00	82.4 - 2.00	82.6 - 2.00	82.6 – 2.00	82.8 – 2.00	82.6 – 2.00	83.0 – 2.00	82.7 – 2.00	83.1 – 2.00
**Cell dimensions**											
**a (Å)**	127.0	127.0	127.2	127.2	127.4	127.3	127.5	127.3	127.6	127.5	127.8
**b (Å)**	46.6	46.6	46.7	46.7	46.7	46.7	46.7	46.7	46.8	46.8	46.8
**c (Å)**	172.8	172.9	173.4	173.5	173.9	173.8	174.4	173.9	174.7	174.1	174.9
**β (°)**	108.3	108.3	108.3	108.2	108.3	108.2	108.2	108.2	108.2	108.2	108.2
**No. Observations**	214907 (15647)	215330 (15653)	215898 (15609)	215805 (15623)	215589 (14888)	215268 (14873)	215641 (14775)	215058 (14794)	216104 (14796)	215297 (14768)	216335 (14487)
**Unique reflections**	65500 (4626)	65572 (4632)	65895 (4619)	65997 (4662)	66183 (4433)	66172 (4451)	66457 (4423)	66250 (4440)	66726 (4444)	66424 (4450)	66960 (4455)
**$CC_{1/2}$ (%)**	99.7 (95.4)	99.7 (94.4)	99.5 (89.4)	99.6 (83.1)	99.4 (64.5)	99.5 (52.8)	99.3 (28.9)	98.6 (23.1)	98.1 (10.3)	96.6 (6.8)	90.3 (2.7)
**I/sig(I)**	12.2 (4.3)	12.2 (4.1)	10.9 (3.1)	10.0 (2.8)	8.3 (1.6)	7.7 (1.7)	6.4 (1.0)	6.4 (1.4)	5.2 (0.8)	5.6 (1.2)	4.3 (0.8)
**Multiplicity**	3.3 (3.4)	3.3 (3.4)	3.3 (3.4)	3.3 (3.4)	3.3 (3.4)	3.3 (3.3)	3.2 (3.3)	3.2 (3.3)	3.2 (3.3)	3.2 (3.3)	3.2 (3.3)
**Refinement**											
**Rwork (%)**	15.4	17.7	18.6	19.1	20.4	20.7	22.4	21.9	24.4	23.1	26.2
**Rfree (%)**	17.9	19.7	20.6	21.2	22.4	22.7	24.4	24.0	26.7	25.2	28.3
**Mean isotropic B-factor ($Å^2$)**	27.8	27.7	30.3	29.5	36.6	31.2	40.1	30.1	34.3	29.5	30.3

Table S2.2: Assessment of final macromolecular geometry for the refined coordinate model corresponding to the first dataset, as reported by *Molprobity4 (Chen et al., 2010).* Since for the coordinate models corresponding to the higher dose datasets, only rigid-body refinement was performed on the refined initial dataset coordinate model coupled with the higher dataset merged structure factor amplitudes, the reported geometry statistics were conserved for the higher dose datasets.

No. non-H atoms	7358
Protein	6686
Water	644
Other	28
RMSD bond length (Å)	0.006
RMSD bond angle (°)	0.998
Ramachandran analysis	
Favoured (%)	98.4
Outliers (%)	0.00
Allowed (%)	1.60
Rotamer outliers (%)	0.28
All atom clash-score	1.78

Supplementary 2 References

Chen, V. B., Arendall, W. B., 3rd, Headd, J. J., Keedy, D. A., Immormino, R. M., Kapral, G. J., Murray, L. W., Richardson, J. S. & Richardson, D. C. (2010). *Acta Cryst.* D**66**, 12-21.

Fuentes-Montero, L., Parkhurst, J., Winter, G., Waterman, D., Gildea, R., Brewster, A., Hattne, J., Sauter, N. & Evans, G. (2014). *Acta Cryst. A* **70**, C1440.

McCoy, A. J., Grosse-Kunstleve, R. W., Adams, P. D., Winn, M. D., Storoni, L. C. & Read, R. J. (2007). *J. Appl. Cryst.* **40**, 658-674.

Murshudov, G. N., Skubák, P., Lebedev, A. a., Pannu, N. S., Steiner, R. a., Nicholls, R. a., Winn, M. D., Long, F. & Vagin, A. a. (2011). *Acta Cryst.* D**67**, 355-367.

Ravelli, R. B. & McSweeney, S. M. (2000). *Structure* **8**, 315-328.

Southworth-Davies, R. J., Medina, M. A., Carmichael, I. & Garman, E. F. (2007). *Structure* **15**, 1531-1541.

Winn, M. D., Ballard, C. C., Cowtan, K. D., Dodson, E. J., Emsley, P., Evans, P. R., Keegan, R. M., Krissinel, E. B., Leslie, A. G. W., McCoy, A., McNicholas, S. J., Murshudov, G. N., Pannu, N. S., Potterton, E. a., Powell, H. R., Read, R. J., Vagin, A. & Wilson, K. S. (2011). *Acta Cryst.* D**67**, 235-242.

Zeldin, O.B., Gerstel, M., Garman, E.F. (2013) *J. Appl. Cryst.* **46**, 1225-1230.

Zeldin, O.B., Brockhauser,S., Bremridge, J., Holton, J.M., Garman, E.F. (2013) *Proc. Natl. Acad. Sci. USA* **110**, 20551-20556.

Supplementary material 3: Tyr –OH versus -C_ζ correlation analysis

To verify whether published reports of negative $F_{obs}(n) - F_{obs}(1)$ difference density at a small subset of Tyr residues (Tyr-330 in myrosinase (Burmeister, 2000), Tyr-63 in TRAP (Bury *et al.*, 2016)) were compatible with a model of radiation-induced ring displacement, D_{loss} values for Tyr -OH have been compared directly with those for covalently bound Tyr-C_ζ atoms. Here, linear regression fitting has been restricted to structures deemed to contain statistically valid sample sizes of atoms (> 60 kDa).

For myrosinase, at the highest dose analysed (Fig. S3.1a) a high positive correlation exists between Tyr-OH and -C_ζ D_{loss}, (linear R^2: 0.72 - 0.82 for dose range), which is of the order of both the Asp -C_γ & -$O_{\delta1}$ (Fig. S3.1c) and Glu -C_δ & -$O_{\varepsilon1}$ (Fig. S3.1e) (with linear R^2: 0.81 - 0.91 and 0.83 - 0.91 respectively for dose range). The observed high correlations for Asp and Glu correspond here to full oxidative cleavage of the carboxylate. The comparably high correlation between D_{loss} for Tyr-C_ζ and Tyr-OH supports a hypothesis of radiation-induced disordering of the overall tyrosyl aromatic ring, fixed as a covalently bound unit. For explicit cleavage of the phenolic C–O bond, a lower correlation would be anticipated, similarly to those reported between the Asp -C_γ & -C_β (Fig. S3.1b) and Glu -C_δ & -C_γ (Fig. S3.1d) atoms (linear R^2: 0.11 - 0.23 and 0.42 - 0.59 respectively across the dose range). These reduced correlations are expected since the Asp-C_β and Glu-C_γ atoms are not predicted to be cleaved during side-chain decarboxylation.

Similarly, for the TRAP damage series, the Asp -C_γ & -$O_{\delta1}$ and Glu -C_δ & -$O_{\varepsilon1}$ D_{loss} behaviour was more correlated than that of Asp -C_γ & -C_β and Glu -C_δ & -C_γ (Fig. S3.2 a-d). For TRAP, the Tyr -OH and -C_ζ behaviour was only weakly correlated across the large dose range studied (1.3 - 25.0 MGy, (Bury *et al.*, 2016)) (Fig. S3.2e). We suggest here that the low correlation observed for Tyr is a consequence of the 11-fold symmetry around each TRAP ring. Each of the two TRAP rings within the asymmetric unit contains 11 symmetry-related copies of a single Tyr residue (Tyr-63), with all of these predicted to exhibit similar D_{loss} values due to the conserved local protein environment (binding interactions, solvent accessibility) for each Tyr-63 residue around a TRAP ring. Consequently, the Tyr scatter plot may not contain an adequate sampling of Tyr residues for linear regression analysis in TRAP.

In contrast, other proteins (malate dehydrogenase (Fioravanti *et al.*, 2007), acetylcholinesterase (Weik *et al.*, 2000), and phosphoserine aminotransferase (Dubnovitsky *et al.*, 2005)) exhibited negligible D_{loss} correlation between Tyr -OH and -C_ζ atoms (Fig. S3.3 & S3-4). However, no Tyr-OH groups were flagged as radiation-sensitive within these structures (Fig. 3, overall Tyr-OH ranks: 42, 36 and 54 for these proteins respectively, with the highest individual Tyr-OH positions being above 290, 138 and 235 at all tested doses). Consequently, a correlation would not be expected between -OH and -C_ζ, with noise dominating at low D_{loss} values. In summary, whereas the correlation between Tyr -OH and -C_ζ atom electron density loss is inconsistent between the investigated proteins, in a subset of cases for which negative $F_{obs}(n) - F_{obs}(1)$ map peaks have been detected near Tyr-OH groups (myrosinase and TRAP), a positive correlation is present.

Figure S3.1 (a-e) Scatter plots to compare D_{loss}(atom) behaviour for atoms within the same residue side-chains, for the highest dose myrosinase crystal dataset (Burmeister, 2000). The linear coefficient of determination is computed for each scatter plot.

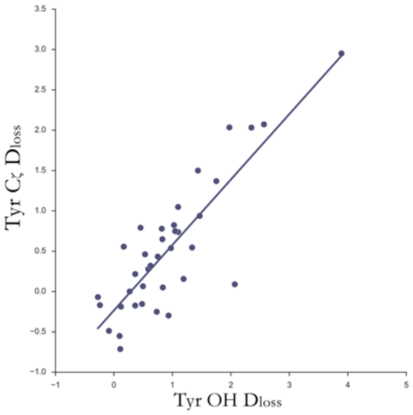

(a) R^2: 0.72

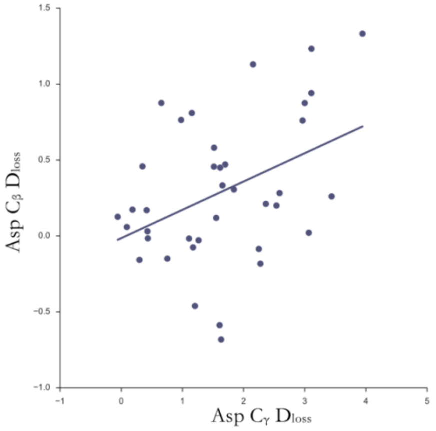

(b) R^2: 0.17

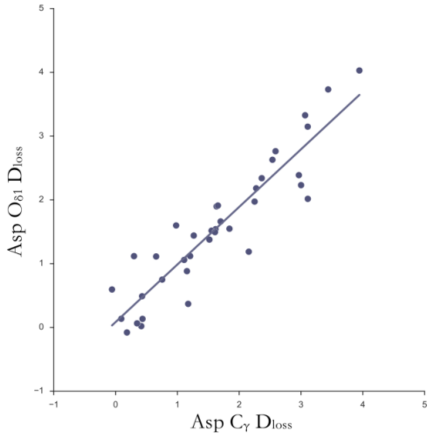

(c) R^2: 0.85

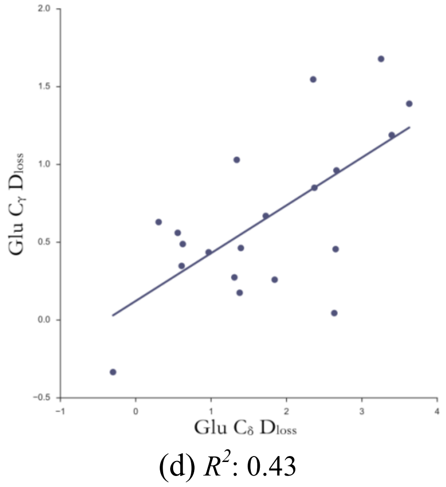

(d) R^2: 0.43

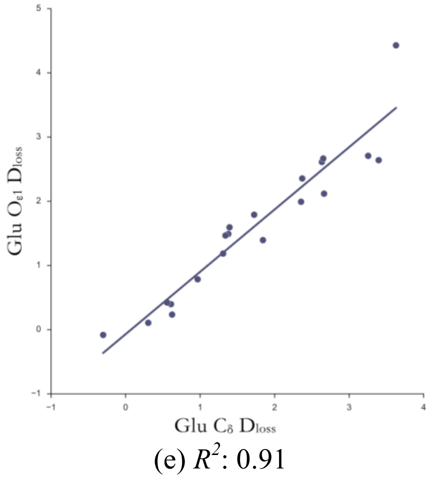

(e) R^2: 0.91

Figure S3.2. (a-e) Scatter plots to compare D_{loss}(atom) behaviour for atoms within the same residue side-chains, for dataset 5 (11.6 MGy) of the TRAP crystal damage series (Bury *et al.*, 2016). The linear coefficient of determination is computed for each scatter plot.

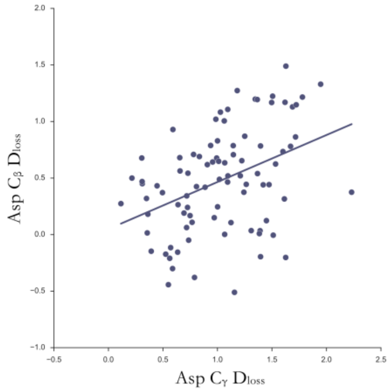

(a) R^2: 0.17

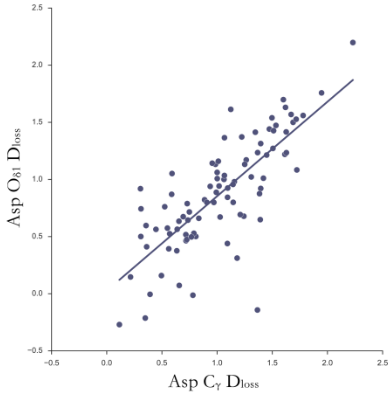

(b) R^2: 0.6

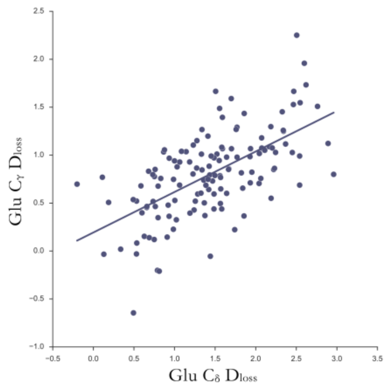

(c) R^2: 0.38

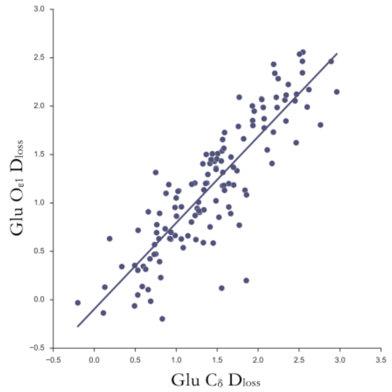

(d) R^2: 0.75

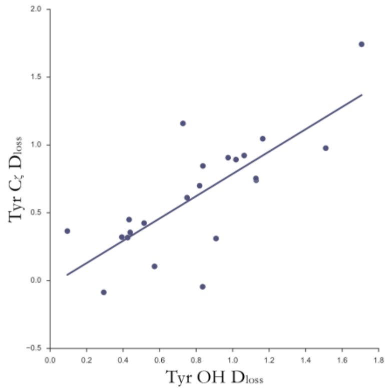

(e) R^2: 0.58

Figure S3.3. Scatter plot to compare D_{loss}(atom) behaviour for Tyr-OH and –C_ζ atoms within the same residue side-chains, for reported doses of (a) 4.6 MGy and (b) 8.2 MGy for the malate dehydrogenase crystal damage series (Fioravanti et al., 2007). R^2 values of (a) 0.21 and (b) 0.17 have been computed for the linear fits in each scatter plot.

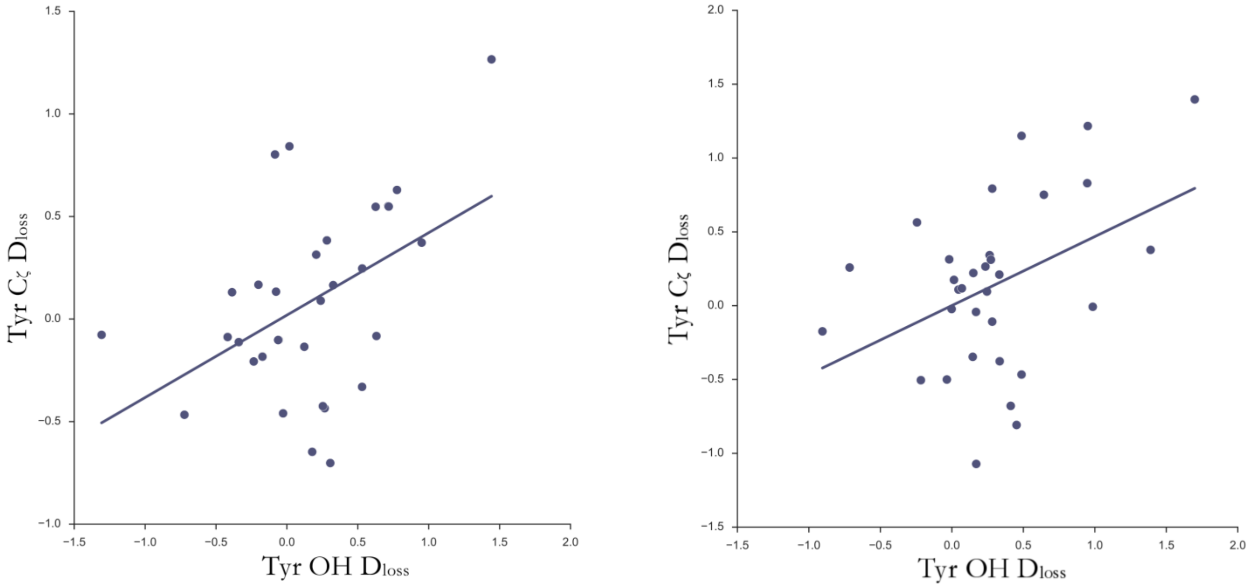

(a) R^2: 0.21 (b) R^2: 0.17

Figure S3.4. The linear coefficient of determination (R-squared) quantifying the linear correlation between D_{loss} for selected atom pairs for large protein structures (> 60 kDa). Box plots illustrate the variation in R-squared value between each dataset for each separate damage series.

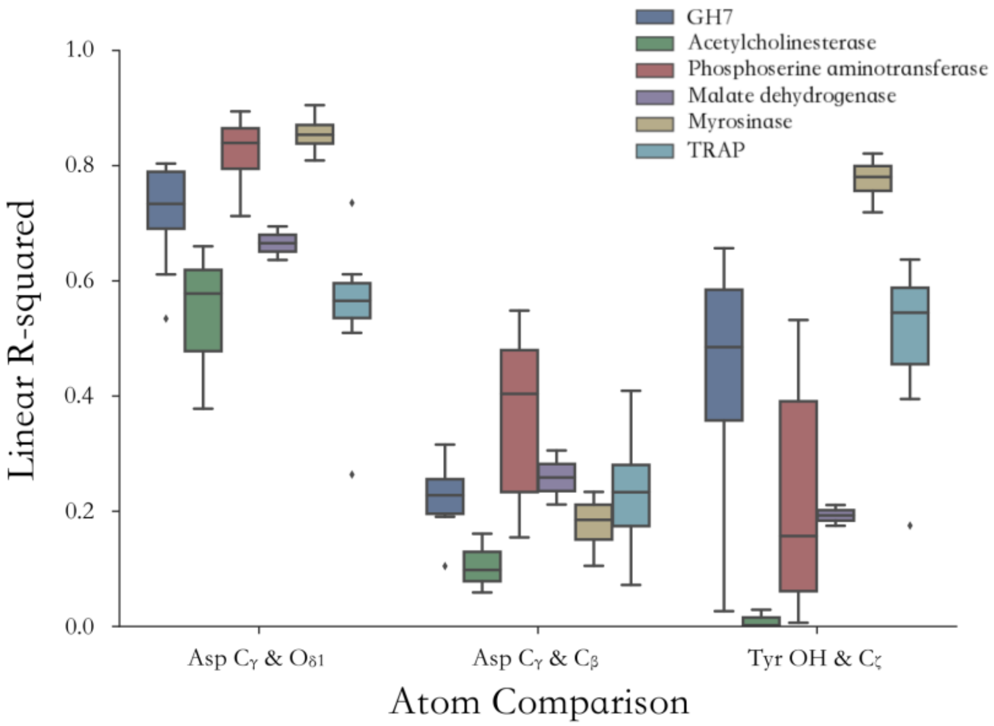

Supplementary 3 References

Burmeister, W. P. (2000). *Acta Cryst.* D**56**, 328-341.

Bury, C. S., McGeehan, J. E., Antson, A. A., Carmichael, I., Gerstel, M., Shevtsov, M. B. & Garman, E. F. (2016). *Acta Cryst.* D**72**, 648-657.

Dubnovitsky, A. P., Ravelli, R. B., Popov, A. N. & Papageorgiou, A. C. (2005). *Protein Sci.* **14**, 1498-1507.

Fioravanti, E., Vellieux, F. M., Amara, P., Madern, D. & Weik, M. (2007). *J Synchrotron Rad.* **14**, 84-91.

Weik, M., Ravelli, R. B., Kryger, G., McSweeney, S., Raves, M. L., Harel, M., Gros, P., Silman, I., Kroon, J. & Sussman, J. L. (2000). *Proc. Natl. Acad. Sci. USA* **97**, 623-628.
